# Inducible Mosaic Cell Labeling Provides Insights Into Pancreatic Islet Morphogenesis

**DOI:** 10.3389/fcell.2020.586651

**Published:** 2020-09-25

**Authors:** Julia Freudenblum, Dirk Meyer, Robin A. Kimmel

**Affiliations:** Institute of Molecular Biology/CMBI, University of Innsbruck, Innsbruck, Austria

**Keywords:** pancreas, zebrafish, mosaic cell labeling, islet, beta cell, diabetes, filopodia, morphogenesis

## Abstract

Pancreatic islets, discrete microorgans embedded within the exocrine pancreas, contain beta cells which are critical for glucose homeostasis. Loss or dysfunction of beta cells leads to diabetes, a disease with expanding global prevalence, and for which regenerative therapies are actively being pursued. Recent efforts have focused on producing mature beta cells *in vitro*, but it is increasingly recognized that achieving a faithful three-dimensional islet structure is crucial for generating fully functional beta cells. Our current understanding of islet morphogenesis is far from complete, due to the deep internal location of the pancreas in mammalian models, which hampers direct visualization. Zebrafish is a model system well suited for studies of pancreas morphogenesis due to its transparency and the accessible location of the larval pancreas. In order to further clarify the cellular mechanisms of islet formation, we have developed new tools for *in vivo* visualization of single-cell dynamics. Our results show that clustering islet cells make contact and interconnect through dynamic actin-rich processes, move together while remaining in close proximity to the duct, and maintain high protrusive motility after forming clusters. Quantitative analyses of cell morphology and motility in 3-dimensions lays the groundwork to define therapeutically applicable factors responsible for orchestrating the morphogenic behaviors of coalescing endocrine cells.

## Introduction

During organ formation, differentiating cells organize into complex structures that correspond with their physiologic roles. Intricate forms emerge through mechanisms that include differential adhesion, dynamic motility, and cell displacements. The vertebrate pancreas consists of digestive hormone-secreting acinar cells, a ductal system and the endocrine islets. The islets, clustered endocrine cells enveloped within the exocrine tissue, develop from progressive coalescence of differentiating progenitor cells that emerge from the ductal epithelium. While previous work suggested that progenitors actively migrate through the pancreatic mesenchyme during islet morphogenesis (Puri and Hebrok, 2007; Kesavan et al., 2014; Pauerstein et al., 2017), recent evidence reveals that clustering occurs in proximity to the pancreatic duct, either concurrent with cell exit (Sharon et al., 2019), or following movements along the ductal epithelium (Nyeng et al., 2019). Imaging studies *in vivo* and in explants have reported formation of dynamic protrusions in endocrine cells (Bechard et al., 2016; Bankaitis et al., 2018; Freudenblum et al., 2018), but their role in islet formation has not been fully defined.

Clarification of the mechanisms of islet formation requires visualization of active cell motility *in vivo*, for which the transparent zebrafish provides an ideal model system. The architecture and physiology of the zebrafish pancreas is highly similar to that of mammals, and a conserved set of genes regulates pancreatic cell type specification and differentiation (Prince et al., 2017). During the “second wave” of islet formation, endocrine progenitors emerge from the duct and cluster to form secondary islets, which in zebrafish initiates at around 5 days post fertilization (dpf) (Wang et al., 2011). Secondary islet development progresses slowly over subsequent weeks, in a stochastic, non-stereotypic manner (Parsons et al., 2009; Freudenblum et al., 2018). Beyond 8–10 dpf, the pancreas becomes increasingly difficult to visualize in the zebrafish due to increasing body wall thickness and contortions of the gut and pancreas (Freudenblum et al., 2018). However, as differentiation of endocrine cells is regulated by Delta-Notch signaling, their rate of appearance can be enhanced by treatment of early larvae with Notch inhibitors (Parsons et al., 2009; Kimmel and Meyer, 2016). Previous studies showed that the subsequent clustering of islet cells, which can be readily imaged *in vivo*, resembles the assembly of later forming, naturally occurring secondary islets (Freudenblum et al., 2018).

In studies of pancreas development in zebrafish, defined promoters can direct expression to pancreatic progenitors as well as exocrine tissue, duct, and islet cell types (Kimmel and Meyer, 2010; Prince et al., 2017). Inducible Cre-recombinase based systems for cell labeling in pancreas have been implemented for lineage tracing and fate mapping (Wang et al., 2015; Singh et al., 2017). Fewer tools are available for investigating cell dynamics, which requires labeling of membrane or cytoskeletal elements to reveal cell contours and fine details of cell morphology. To study motility and migration during early development in zebrafish, techniques such as DNA or RNA injection, or cell transplantation, are used to create mosaics in which single cells can be distinguished (Andersen et al., 2010; Boutillon et al., 2018), but such approaches are not easily applied to studying events during later developmental stages. The Gal4/UAS system can be used to label cell populations with spatial control provided by tissue-specific Gal4 expression (Weber and Köster, 2013), and using a Gal4ER (Estrogen Receptor binding domain) fusion protein provides additional tamoxifen-responsive temporal control (Gerety et al., 2013; Akerberg et al., 2014). Although demonstrated to be effective for inducible, spatially restricted transgene expression, Gal4ER and a related KalTA4ER have until now been infrequently implemented (Calzolari et al., 2014; Laux et al., 2017).

In this work, we develop new tools for studying cell motility during islet morphogenesis. We show that a 5 kb *neurod* promoter, previously used for studies of the zebrafish nervous system (Mo and Nicolson, 2011; Cook et al., 2019), also directs robust expression to pancreatic islet cells. A *neurod:memKate* transgenic line highlights morphology of all endocrine cell types. We further combined the *neurod* promoter with the tamoxifen-responsive Gal4ER fusion protein in an inducible system providing spatial and temporal control of gene expression. We demonstrate the rapid responsiveness and tightly regulated induction of *neurod:Gal4ER* for activating UAS responder lines, and we apply this approach to precisely characterize motility and morphology of clustering islet cells. Time lapse studies reveal heterogeneous protrusive behaviors with stable cell-cell connections leading to directed cell translocations. Mosaic cell labeling permitted analysis of single cell morphology in three dimensions, which established that cell dynamics are maintained as endocrine cells incorporate into clusters. The genetic and quantitative methods reported here can help to define molecular regulators of islet morphogenesis, and be further applied in broader developmental contexts.

## Results

### Tight Apposition of Pancreatic Tissue Compartments

Recently published work asserts that differentiated endocrine progenitors remain attached to one another and to the duct as they cluster to form bud-like islets (Sharon et al., 2019). To explain a lack of movement away from the duct, we hypothesized that close apposition of pancreatic cell types may represent a physical barrier that restricts cell movements. To define the spaces occupied by pancreatic tissue compartments, we generated triple-transgenic zebrafish in which endocrine, duct and exocrine compartments are labeled by cytoplasmically localized fluorescent reporters. A previously generated line using the far red E2-Crimson fluorophore labels exocrine tissue (*ela:*E2-Crimson, Schmitner et al., 2017), and can be distinguished from a DsRed transgene expressed in endocrine cells (*pax6b:DsRed*). Ductal progenitors are labeled by the *Tp1:GFP* transgene (Parsons et al., 2009). To visualize secondary islet cells, we applied our previously validated method to trigger early endocrine cell differentiation using an inhibitor of Notch signaling (Freudenblum et al., 2018).

**FIGURE 1:**
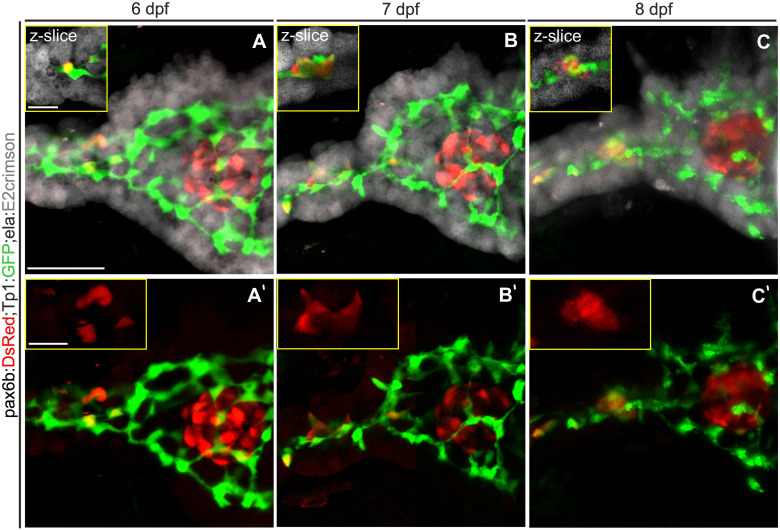
The exocrine pancreas envelops the duct and the endocrine pancreas. **(A–C)** Z-projections of confocal stacks showing endocrine (*pax6b:DsRed*, red), exocrine *(ela:E2crimson*, gray) and ductal *(Tp1:GFP*, green) pancreatic compartments. Single slice close-ups (yellow boxes) highlight the close apposition of the exocrine tissue to the duct and endocrine cells. **(A’–C’)** Corresponding 2-channel images to highlight Notch-inhibitor induced secondary islet cells which move together (insets), but remain near the duct when imaged at 24 h intervals between 6 and 8 dpf. Scale bars: 50 μm; insets, 10 μm.

By imaging through confocal stacks, a close apposition between the three tissue compartments can be observed (Figures 1A–C). At the head of the pancreas, the principal islet is tightly apposed by exocrine tissue and intervening ductal cells. In the pancreatic tail, the ductal contours are closely matched by those of exocrine tissue. In images collected at 24 hour intervals, clustering of secondary islet cells could be observed (Figures 1A’–C’). The cells changed morphology and moved together, without moving away from the duct. These constricted spatial relationships are consistent with a model of endocrine cell clustering by movement along the duct, without long-distance migration through the mesenchyme.

### A Tool for Highlighting Cell Morphology During Islet Formation

To gain further insight into cellular mechanisms of islet formation, we generated novel transgenic tools for better visualization of cellular morphology and motility. The transcription factor *neurod* plays a critical and conserved functional role in endocrine cell differentiation in zebrafish and mammals (Naya et al., 1997; Flasse et al., 2013), and a previously generated BAC transgenic line, *TgBAC(NeuroD:EGFP)nl1* [hereafter referred to as *BAC(nd:EGFP)*], is expressed in the central nervous system and lateral line, as well as in pancreatic endocrine cells (Dalgin et al., 2011; Kimmel et al., 2011; Thomas et al., 2012). While the *BAC(nd:EGFP*) transgenic line is extremely useful for visualizing the early emerging endocrine cells, modifying this transgene for studies of cell dynamics would be technically challenging. A 5 kb fragment of the *neurod* promotor was developed for studies of the nervous system (Mo and Nicolson, 2011), but has not previously been validated for studies of endocrine pancreas. To develop a tool for studying membrane dynamics in endocrine cells, we combined this 5 kb *neurod* promoter with a membrane-tagged red fluorescent protein to generate the *nd:memKate* transgene.

**FIGURE 2:**
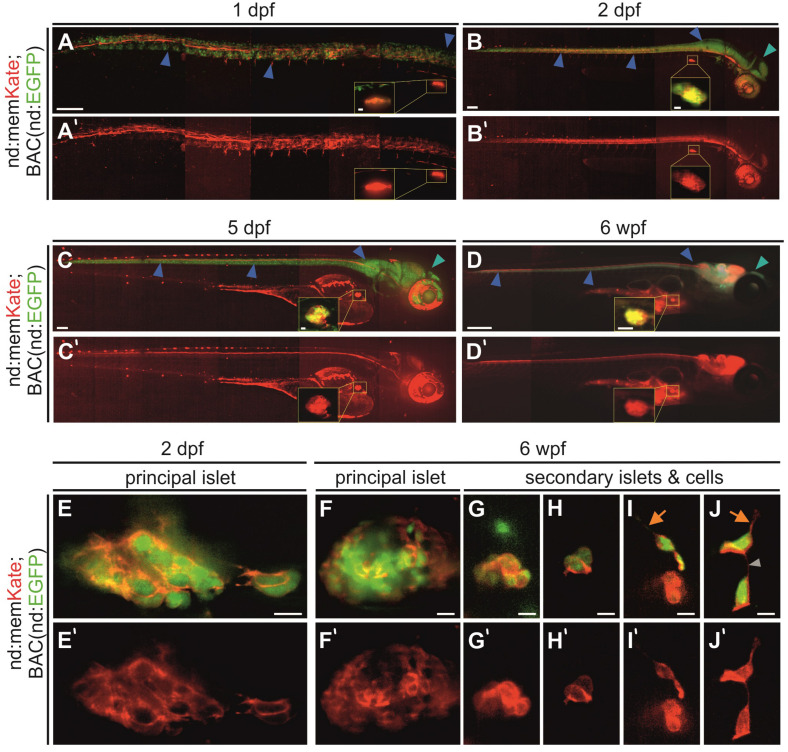
*Neurod*-promoter driven transgene expression in the nervous system and pancreas. **(A–D)** *In vivo* Kate (red) and EGFP (green) expression in the neural tube, head and pancreatic principal islet at the stages indicated. The yellow insets show a higher magnification view of the principal islet. Full images were assembled by stitching together partially overlapping regions. **(E–J)** High magnification overlay of *neurod*-promotor (red) and *neurod*-BAC (green) expression in the pancreatic principal and secondary islets of fixed and dissected samples at 2 dpf and 6 wpf. Blue arrowhead, neural tube; cyan arrowhead, head region; orange arrow, protrusion; gray arrowhead, cell-cell connection. Scale bars: **(A–C)**, 100 μm; inset, 10 μm; **(D)**, 500 μm; inset, 250 μm; **(E–J)**, 10 μm. **(A’–J’)** Single channel *nd:memKate* expression.

To confirm that this promoter fragment drives endocrine as well as nervous system expression, we analyzed memKate expression in embryos also containing *BAC(nd:EGFP)*. Overall, the *neurod* promotor-driven expression pattern correlated with the expression pattern of the BAC transgene during embryogenesis (Figures 2A–D). Compared to BAC transgenes insulated by extensive genetic sequences thought to minimize position effects, expression driven by promoter fragments can be variable depending on site of integration (Beil et al., 2012). In some cases, they may not recapitulate the full gene expression pattern. In examining the F1 generation, we noted variability in strength of pancreatic islet versus nervous system expression. For further experiments we selected and maintained lines showing consistent and strong endocrine pancreas expression.

In established *nd:memKate* transgenic lines, *neurod*-promoter driven expression in the central nervous system and the principal islet of the pancreas was consistent through larval and juvenile stages (Figures 2A–D). With higher magnification imaging of the principal islet at embryonic (2 dpf) and juvenile (6 weeks post fertilization, wpf) stages, we observed memKate labeled cell membranes of cells expressing cytoplasmic EGFP from *BAC(nd:EGFP)* (Figures 2E,F). In zebrafish, secondary islet cells begin to differentiate after 5 dpf and form clusters that progressively increase in size. To confirm that the *neurod* promoter also drives expression in secondary islet cells, we examined dissected pancreata from 6 week old *nd:memKate;BAC(nd:EGFP)* double transgenics (Figures 2G–J). The membrane targeted memKate co-localized with EGFP in the principal and secondary islets. In loosely associated secondary islet cells, the memKate highlighted cell contours as well as protrusions and fine cell-cell connections (Figures 2I,J, arrows), which are proposed to play a functional role in the assembly process (Freudenblum et al., 2018; Nyeng et al., 2019).

We previously analyzed fine cellular extensions formed by early beta cells based on *mnx1:memGFP* transgene expression (Freudenblum et al., 2018). The newly generated *nd:memKate* transgene enabled us to examine cell motility and assembly additionally in further differentiated endocrine cells. Within gcga:GFP-expressing alpha cells and sst:GFP-expressing delta cells, as well as in ins:GFP-expressing beta cells, protrusions extending > 1 cell diameter in length were detected in loosely clustered cells in the process of forming tighter aggregations, and within cell clusters (Supplementary Figure S1). Thus, protrusion formation is not limited to the beta cell lineage, and is a behavior that is maintained as cells differentiate.

### Emerging Islet Cells Remain Near the Duct

**FIGURE 3:**
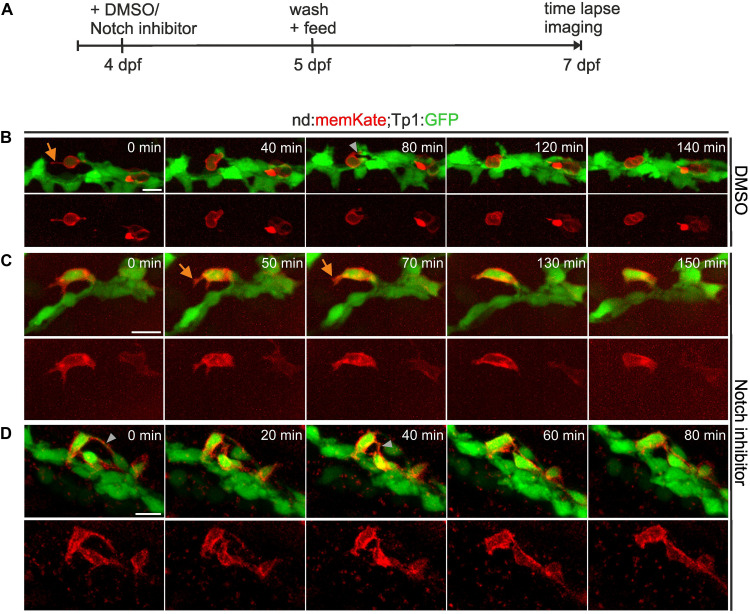
Secondary islet cells remain near the duct. **(A)** Schematic of experimental approach. **(B–D)** Z-projections of confocal image stacks in control **(B)** and Notch inhibitor-treated **(C,D)** *Tp1:GFP;neurod:memKate* transgenics at 7 dpf, acquired by time lapse imaging at the times indicated (min = minutes). Single channel images of *neurod:memKate* expression are shown below each 2-color panel. Orange arrow, protrusion; gray arrowhead, cell-cell connection. Scale bars, 10 μm.

To further examine cell movements during islet formation, we used the *Tp1:GFP* transgenic line in combination with the *nd:memKate* transgene, and followed naturally arising as well as induced secondary islet cells at 7 dpf for up to 2.5 h by time-lapse imaging (Figure 3A). *nd:memKate*-expressing cells maintained contact with the duct via cytoplasmic extensions (Figures 3B–D). Narrow bridges between endocrine cells could be observed, which changed over time (Figure 3D). Endocrine cells formed small clusters at the periphery of the duct (Figure 3D), while migration away from the duct was not observed (*n* = 14 movies). To support these findings, we extended the live imaging up to 4 days. Following induction of endocrine cell differentiation by Notch inhibition at 4 dpf, we imaged the pancreas at 6, 7, 8, and 9 dpf using our catch-and-release approach (Freudenblum et al., 2018) (Supplementary Figure S2A). Induced secondary islet cells extended protrusions, and changed position over time to form linear arrays and small clusters, while remaining in contact with the duct. Even over these longer time intervals, we did not observe movement of cells away from the duct (Supplementary Figure S2B, *n* = 6 embryos).

**FIGURE 4:**
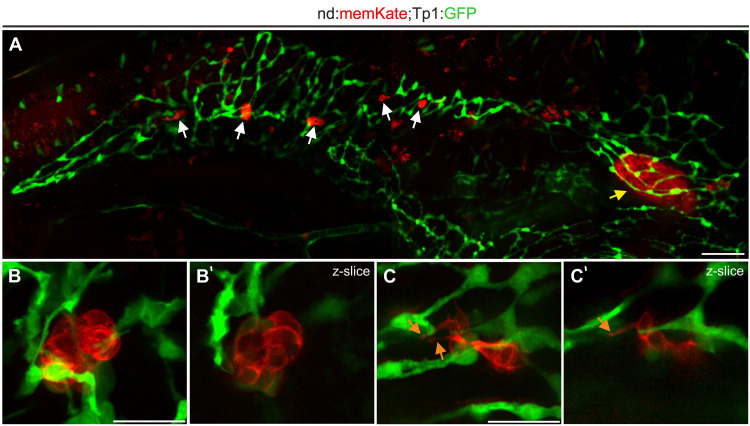
Naturally occurring secondary islet cells and clusters remain duct-associated. **(A)** Pancreas of *Tp1:GFP;neurod:memKate* double-transgenic imaged at 2 wpf. Indicated are the principal islet (yellow arrow) and secondary islets (white arrows). **(B,C)** Higher magnification views of secondary islets from samples as in **(A)**, showing directly adjacent ductal tissue (green). Maximum intensity projections **(B,C)** and single slice images **(B’,C’)** of secondary islets. Orange arrow, protrusion. Scale bars: **(A)**, 50 μm; **(B,C)**, 20 μm.

To visualize naturally occurring secondary islets in relation to the duct, we imaged 2 week old *neurod:memKate*;*Tp1:GFP* transgenics. The principal islet and secondary islets are surrounded by the ductal plexus (Figures 4A,B) and secondary islet cells show protrusions which are in contact with ductal cells (Figure 4C and Supplementary Movie S1). These results are consistent with recent data from mouse, demonstrating that also in zebrafish, islet cells coalesce in close proximity to the duct.

### Inducible Endocrine Cell Transgene Expression

**FIGURE 5:**
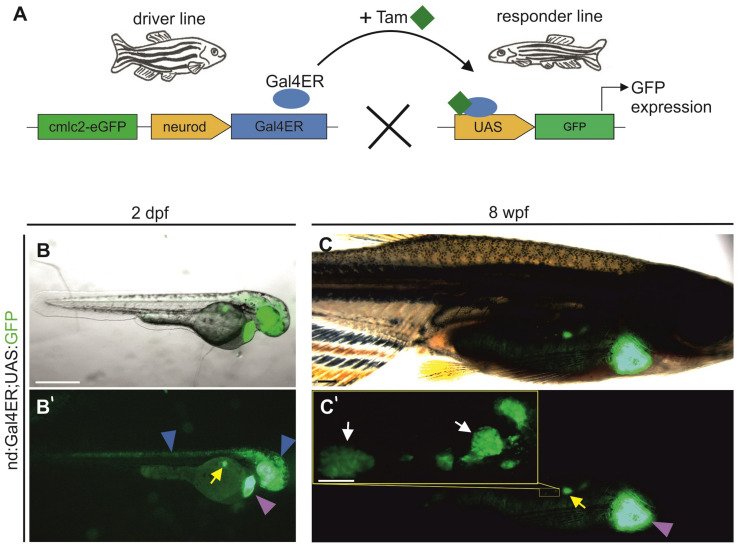
*neurod* promoter-driven inducible transgene expression. **(A)** Schematic of Gal4ER-mediated transgene activation. The driver line expresses Gal4ER (blue oval). Upon Tam (green squares) treatment, Gal4ER activates the UAS-linked responder line (shown is UAS:GFP). **(B,C)** Visualization of GFP expression *in vivo* in Tg(*nd:Gal4ER;UAS:GFP*) embryos (2 dpf) and juveniles (8 wpf), following Tam treatment. Images shown are overlay of GFP and a bright field image. **(B’,C’)** Corresponding image showing GFP expression alone. Blue arrowheads indicate GFP expression in the nervous system, yellow and white arrows the expression in the principal islet and secondary islet cells, respectively. Yellow inset **(C’)** shows secondary islet cells and secondary islets at higher magnification. Purple arrowhead indicates the heart transgenesis marker (*cmlc2-EGFP*). Scale bars: **(B)**, 500 μm; **(C’**, inset), 200 μm.

While the *nd:memKate* transgene highlights cell membranes of the clustering endocrine cell population, it is difficult to discern morphologies of individual cells (Supplementary Figure S1). Quantitative studies of cell dynamics are facilitated by sparse labeling of cells, which in turn enables identification of regulating factors that act cell autonomously. To pursue this end, we adapted the inducible Gal4ER/UAS system to label endocrine cells, by first generating a driver line which expresses Gal4ER under control of the *neurod* promoter (*nd:Gal4ER*). Functionality and specificity were tested by crossing *nd:Gal4ER* transgenics with *UAS:GFP* transgene-containing fish (Figure 5A). Following overnight treatment with Tam at 24 h post fertilization (hpf), double transgenic embryos examined at 48 hpf exhibited GFP expression in regions of *neurod* expression, including eye, brain, posterior nervous system, and endocrine pancreas (Figure 5B). We also tested induction in juvenile (8 wpf) fish following exposure to Tam for one hour per day for 3 days. Robust GFP expression was detected in the principal and secondary islets (Figure 5C) and brain structures (not shown), demonstrating functionality of the system at post-larval stages. Double *nd:Gal4ER;UAS:GFP* transgenics did not show fluorescence prior to Tam treatment (*n* = 11), confirming that the system is not leaky (Supplementary Figures S3A,B), while specific GFP expression in pancreas and nervous system was readily detected after Tam treatment (Supplementary Figures S3C,D). As with the *nd:memKate* transgenic, we observed variability in Tam-activated expression. For our studies, we selected *nd:Gal4ER* lines showing strong and consistent Tam-specific induction as tested in combination with the *UAS:GFP* transgene.

To define kinetics of onset and minimal dosage requirements, we treated embryos at 5 dpf with Tam for 1 hour and examined the onset of detectable GFP expression (Supplementary Figures S4A–I). GFP was first observed in the principal islet in 18% of embryos 3 h after the end of treatment with 1 μM Tam. With higher Tam doses (5 μM, 10 μM) more embryos showed expression after 3 h. After 24 h post-treatment, all embryos showed principal islet GFP expression (Supplementary Figures S4J–M and Supplementary Table S1).

**FIGURE 6:**
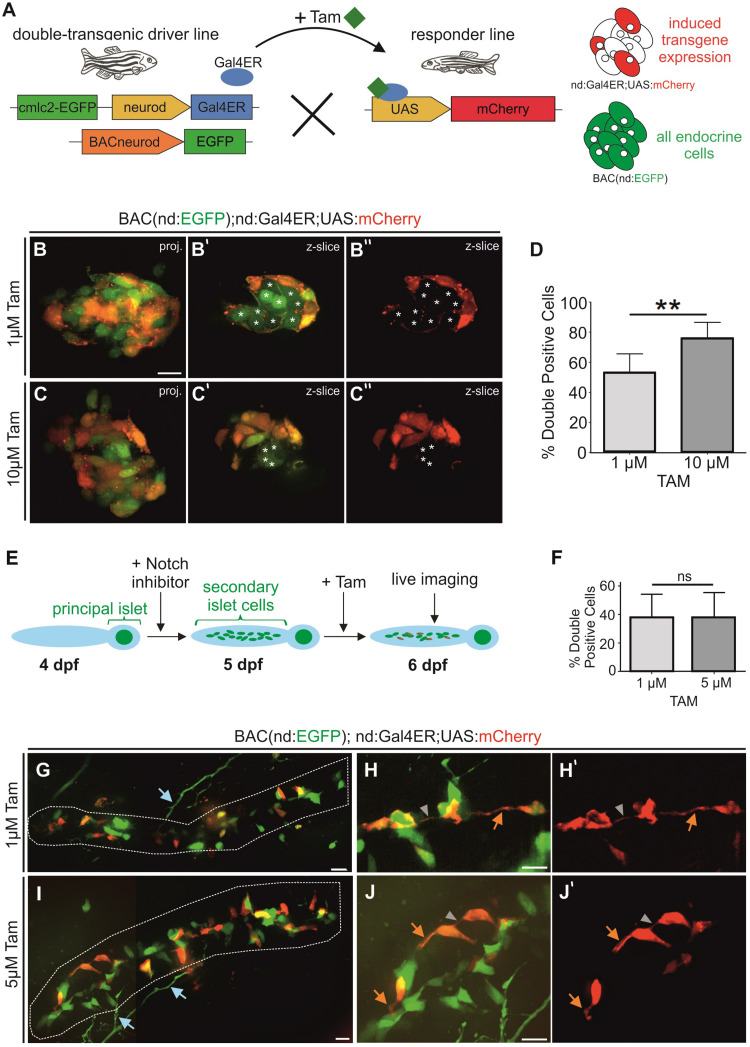
Mosaic labeling of endocrine cells. **(A)** Scheme for examining efficiency of Gal4ER activation. All endocrine cells are labeled by *BAC(nd:EGFP)*, mCherry (red) indicates the expression induced by Tam activation of Gal4ER. **(B,C)** Z-projections and single z-slice **(B’,C”)** images of principal islets of 2 dpf embryos after overnight Tam treatment at the doses indicated. Single z-slice images show GFP/mCherry **(B’,C’)** or mCherry only **(B”,C”)**. Asterisks (*) indicate GFP+/mCherry-cells. White stars mark GFP-only positive cells. **(D)** Quantification of double-positive islet cells relative to the GFP + endocrine cell number of embryos as in **(B,C)** (1 μM *n* = 8, 54%; 10 μM *n* = 9, 76%; *p* = 0.0016; graphed is mean ± s.d.). **(E)** Experiment to mosaically label secondary islet cells after Notch inhibitor-triggering of endocrine cell differentiation. In triple *nd:Gal4ER; UAS:mCherry;BACnd:EGFP* transgenics, a subset of endocrine cells express mCherry after Tam activates Gal4ER activity, while all endocrine cells are labeled by *BAC(nd:EGFP).* **(F)** Percentage (%) of GFP + secondary islet cells that are also mCherry +, after 1 μM and 5 μM Tam treatment overnight (1 μM *n* = 14, 38%; 5 μM *n* = 20, 37%; *p* = 0.9721; graphed is mean ± s.d.). **(G–J)** Maximum intensity projections of representative samples from experiment outlined in **(E)** following Tam treatment at the indicated concentrations, and quantitated in **(F)**. **(G,I)** Secondary islet cells in the posterior pancreas (white outline) at 6 dpf (stitched images). **(H,J)** Close-ups of secondary islet cells. **(H’,J’)** show corresponding single channel images of mCherry expression. Orange arrow, protrusion; gray arrowhead, cell-cell connection; blue arrows, neuronal processes (s.d., standard deviation). Scale bars, 10 μm. **p < 0.01.

To determine the maximal percentage of induction that could be achieved, we generated double transgenics containing *BAC(nd:EGFP)*, to label the whole endocrine cell population, in combination with *nd:Gal4ER* (Figure 6A). We then applied Tam to embryos from a cross of these double transgenics and a homozygous *UAS:mCherry* responder line that were selected for expression of *BAC(nd:EGFP)* and the heart transgenesis marker (*cmlc2:GFP*). Following overnight treatment with 1 μM and 10 μM Tam, we imaged mCherry and GFP expression in the principal islet (Figures 6B,C). 10 μM Tam yielded a higher percentage of GFP+/mCherry+ double positive cells (76%) as compared to 1 μM (54%) (Figure 6D). Tam-responsive expression within Notch inhibitor-induced secondary islet cells varied between embryos within the same treatment groups (Figures 6E,F). Triple transgenics exposed to 1 μM Tam (*n* = 14) showed on average 38 ± 17% double-positive secondary islet cells. Increasing the dose to 5 μM Tam did not significantly change the cell labeling efficiency or variability, with 37 ± 18% double positive cells (*n* = 20) (Figure 6F). The mosaic mCherry labeling revealed cytoplasmic extensions forming intercellular connections (Figures 6G–J). Notch-inhibitor treated samples were more susceptible to toxicity from Tam, and overnight exposure to doses above 5 μm caused more than 50% lethality. Induced mCherry expression within naturally occurring secondary islets at 8 wpf was similarly mosaic (Supplementary Figure S5).

To apply the *nd:Gal4ER* transgene for studies of islet cell morphogenesis, we next developed responder lines containing a UAS response element upstream of a red fluorescent transgene (Tandem-dimer-Tomato, TdT) tagged with an actin-targeting sequence (LifeAct) (Supplementary Figure S6A). We tested the functionality of *UAS:LifeActTdT* lines in combination with a heat shock-inducible Gal4 (*hsp70:Gal4*) transgenic line (Supplementary Figure S6A). Heat-shock treatment of embryos at 1 dpf resulted in robust ubiquitous activation of LifeActTdT expression detectable at 24 after heat shock and persisting until at least 5 dpf (Supplementary Figure S6B,H). In addition, no abnormalities in development resulting from LifeActTdT expression were observed. Actin-rich structures could be identified in various tissues, such as muscle, notochord, skin and nerve cells (Supplementary Figures S6C–G,I–M).

### Dynamic Cell Behaviors Persist During Islet Coalescence

**FIGURE 7:**
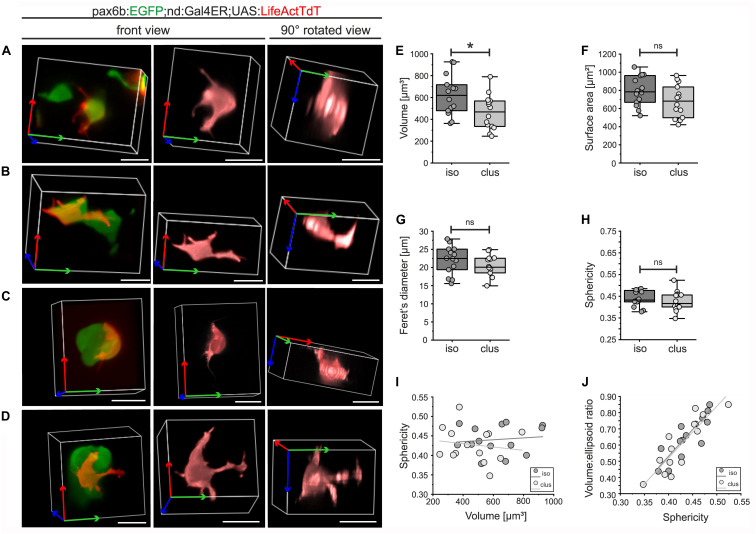
3D morphology of isolated and clustered secondary islet cells. *pax6b:GFP;nd:Gal4ER;UAS:LifeActTdT* larvae were treated by Notch inhibitor at 4 dpf followed by Tam treatment (for details see Supplementary Table S2). Representative single LifeActTdT-labeled cells categorized as isolated **(A,B)** or clustered **(C,D)**, shown in the context of nearby pax6b:GFP+ islet cells (left), as a surface rendering in the original orientation (center), and rotated 90 degrees (right). **(E)** Cell volume is decreased in clustered (clus) as compared to isolated (iso) cells (*t*-test, *p* = 0.02), while surface area, Feret’s diameter and sphericity are similar between the 2 groups (**(F–H)**, see Supplementary Table S3). **(I)** Scatter plot of volume versus sphericity, followed by linear regression analysis, showed no correlation for isolated or clustered cells (slope∼0). **(J)** Volume:ellipsoid ratio versus sphericity showed a positive correlation of these parameters, with no difference between the groups (see Supplementary Table S4). For **(E–J)**, isolated cells *n* = 14; clustered cells *n* = 14. *p < 0.05; ns, not significant. Scale bars, 10 μm.

Complex cell morphologies that arise due to intrinsic molecular and biomechanical processes, coordinated in response to external cues, contribute to the progression of morphogenesis (Mammoto and Ingber, 2010). Mosaic labeling of emerging pancreatic islet cells with actin-targeted fluorescence enabled visualization of fine details of cell morphology and facilitated segmentation of individual cells in three dimensions (3D). To identify features and behaviors that are modulated as cells transition from loosely associated to more tightly clustered aggregates, we analyzed endocrine cells at distinct stages of islet formation. To reveal single endocrine cell morphology, we examined Gal4ER-induced *UAS:LifeActTdt* expression in combination with *pax6b:GFP*, to label all endocrine cells without the potentially confounding labeling of nerve fibers seen with *BAC(nd:EGFP)* (Figures 6G,I). *pax6b:GFP;nd:Gal4ER;UAS:LifeActTdT* triple transgenic embryos were treated sequentially with Notch inhibitor followed by Tam, and individual cells were analyzed. For 3D quantitative morphology measurements, we focused on single cells that were categorized either as isolated or loosely assembled (Figures 7A,B), or within larger (4 or more cells) clusters (Figures 7C,D). Cells in both categories showed diverse and complex morphologies, with extension of broad cytoplasmic protrusions and fine filopodia that varied from straight to flexible and branched (Figures 7A–D, center and right). As cells clustered, volume showed a small but significant decrease, while other parameters of cell size, specifically surface area and Feret’s diameter, varied between cells but overall did not significantly change upon clustering (Figures 7E–G and Supplementary Figure S7; *n* = 14 ‘isolated,’ *n* = 14 ‘clustered’).

Sphericity, a measure of shape complexity, was also similar between the two groups of cells (Figure 7H). Plotting sphericity as a function of volume revealed that cell complexity did not vary with cell size (Figure 7I, R^2^ = 0.014 isolated cells; R^2^ = 0.023, clustered cells), and that the data points from the two categories were intermingled and did not separate into distinct morphotypes. Measures of complexity (sphericity and the cell volume to ellipsoid volume ratio) showed a linear association (R^2^ = 0.704 isolated cells; R^2^ = 0.765, clustered cells) in a continuous distribution, with no segregation of data points from isolated versus clustered cells (Figure 7J).

### Actin Rich Protrusions Mature Into Intercellular Bridges Which Direct Cell Movements

**FIGURE 8:**
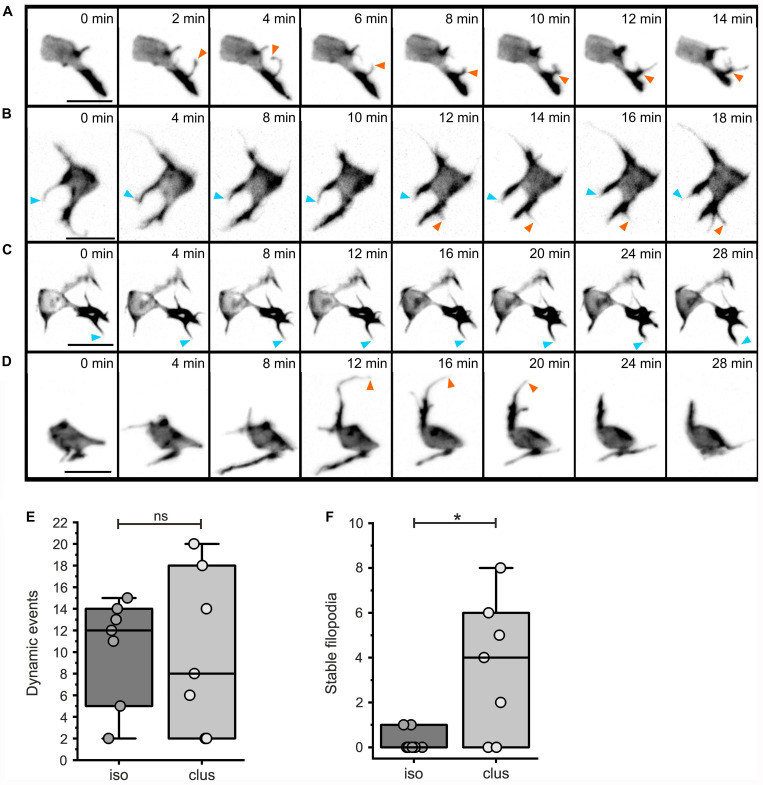
Dynamic protrusive activity of both isolated and clustered cells. Analysis of filopodial dynamics from time lapse series of mosaically labeled secondary islet cells (as in Figure 7; for details see Materials and Methods and Supplementary Tables S2, S5). Z-projections of 3D confocal time-lapse sequences captured at the times indicated, showing isolated **(A,B)** or more clustered **(C,D)** cells. Stable (blue arrowheads), and dynamic (orange arrowheads) protrusions are indicated. Dynamic events were equally frequent in isolated as compared to clustered cells **(E**, *p* = 0.93), while clustered cells show an increased number of stable filopodia **(F**, *p* = 0.016; see Supplementary Table S6). *p < 0.05; ns, not significant. Scale bars, 10 μm.

To further investigate cell dynamics during islet formation, we performed time lapse imaging studies with short (2 minute) time intervals. LifeActTdT expressing cells in either isolated or clustered configurations displayed active extension and retraction of actin-rich filopodia (Figure 8, Supplementary Figure S8, and Movies 2–5). By tracking filopodia appearance and disappearance, as well as extension and retraction, we could define behavior as dynamic or relatively stable. While the number of dynamic protrusive events were similar between isolated and clustered cells (Figure 8E, *p* = 0.933), clustered cells showed a significant increase in the number of less motile filopodia (Figure 8F, *p* = 0.016).

**FIGURE 9:**
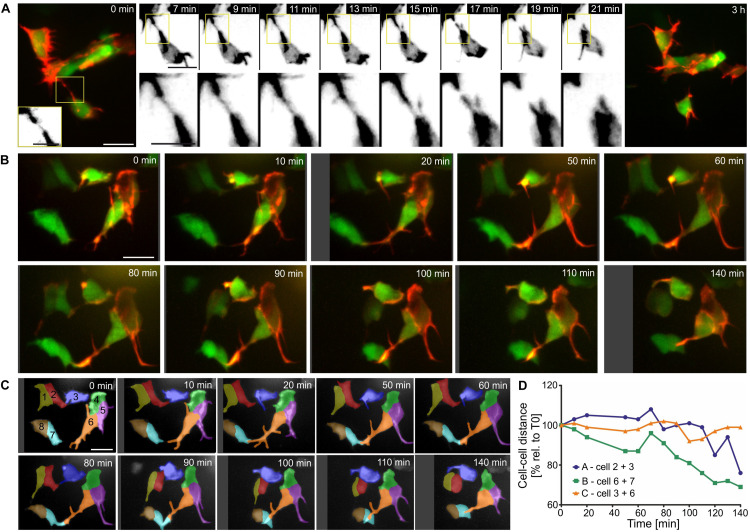
Filopodia form both transient and stable connections. Z-projections of confocal time-lapse series of Tam treated triple Tg*(pax6b:GFP;nd:Gal4ER; UAS:LifeActTdT)* larvae (for details see Supplementary Table S2). **(A,B)** LifeActTdT actin labeling is shown in red or gray, combined with *pax6b:GFP* (green, far left and far right panels in **A)**. **(A)** Example of a transient connection between nearby cells. **(B)** Movements of loosely clustered cells correlate with formation of cell-cell connections. **(C)** Time series shown in **(B)**, with cells segmented and labeled by number and pseudocoloring to facilitate identification in subsequent images and analysis of cell movements. **(D)** Quantification of the center-to-center distance of cells over time show that cells which connected through protrusions (line ‘**A**’ - cells 2 + 3, line ‘**B**’ - cells 6 + 7) move closer together, compared to cells that were not attached to each other (line ‘**C**’ – cells 3 + 6). Scale bars, 10 μm.

Persistent and dynamic protrusive behavior, leading to formation of stable cell-cell connections, may serve as a mechanism for single cells to join nascent islets, and for steady accumulation of cells into increasingly larger clusters. To explore this possibility, we examined protrusions and corresponding cell displacements at high resolution over shorter and longer time intervals. In LifeActTdT labeled cells, fine protrusions showed stronger intensity actin labeling in the proximal regions, and a weaker signal in the tapering distal ends (Figure 9A and Supplementary Figure S9). We observed heterogeneous behaviors in cells making contact through protrusions. In some examples, the meeting of distal tips from opposing filopodial extensions was transient, and did not lead to formation of a stable connection (Figure 9A and Supplementary Figures S9A,C). Contacting filopodia in some cases appeared flexible and not under tension (Supplementary Figure S9B), while in other instances the connection seemed to stretch and break apart (Figure 9A and Supplementary Figures S9A,C). Continued observation over subsequent hours confirmed that the disrupted cell-cell attachment had not been reestablished (Figure 9A and Supplementary Figure S9A). Protrusions in other samples formed stable attachments which matured into intercellular bridges that resulted in cells moving together (Figures 9B–D and Supplementary Figures S10, S11). Interestingly, in following changes in cell-cell distances over time, intervals of increased cell-cell distance were interspersed during an overall trend toward coming together (Figure 9D and Supplementary Figure S11B). This implies a complex sequence of stretching and pulling events. Overall, these studies suggest that islet assembly progresses through stochastic protrusive behaviors which lead to both transient and persistent cell-cell attachments.

## Discussion

In this study we have developed a genetic system for mosaic labeling of the actin cytoskeleton in emerging endocrine cells, to enable detailed examination of cell-cell interactions and directed translocations during islet formation. We demonstrate that the previously reported 5 kb *neurod* promotor (Mo and Nicolson, 2011) is active in pancreatic endocrine cells, and we incorporated this promoter into transgenic lines for studying endocrine cell morphology. Using these novel tools, we show that in zebrafish, differentiated endocrine cells remain adjacent to the duct during islet formation, consistent with recently proposed models in mouse (Sharon et al., 2019). Analysis of 3D morphology and dynamics in nascent islet cells revealed persistent motile and protrusive behaviors, which continued as cells incorporated into clusters. Thus, cells within clusters maintain the capacity to form new contacts with cells in the vicinity, suggesting a mechanism for steady accumulation of cells into increasingly larger aggregates.

Movement of islet progenitors and beta cells has been observed in pancreatic explant studies, and protrusions have been reported in cells emerging from the duct (Puri and Hebrok, 2007; Kesavan et al., 2014; Bechard et al., 2016; Pauerstein et al., 2017; Bankaitis et al., 2018). Based on these studies, it was thought that differentiated endocrine cells first move away from the duct into the mesenchyme and then cluster to form islets (Bakhti et al., 2019). However, recently published work suggests that instead, the differentiated endocrine cells remain in contact with the duct, move together and aggregate to form what were termed peninsulas (Sharon et al., 2019). With our approach using zebrafish larvae, it is possible to visualize not only the whole endocrine cell population in relation to the ductal progenitor compartment, but also the morphology of single endocrine cells in 3D. Consistent with observations from explant studies showing that beta cells produce cytoplasmic protrusions and move along the duct to join other beta cells (Nyeng et al., 2019), our studies revealed that endocrine cells stay in proximity to the duct, and use dynamic protrusions to initiate cell-cell contacts which appear to direct cell coalescence.

To quantitatively analyze relationships between 3D cell morphologies and morphogenic behaviors, which provides a framework for identifying regulating factors, sparse cell labeling is required. For this we implemented the Gal4ER/UAS system. Our newly established *neurod:Gal4ER* line extends the possibilities for inducible gene expression in pancreatic endocrine cells as well as in the nervous system. Transcriptional activation of a UAS responder line was detectable within 3 h of Tam treatment and the expression was highly robust in the endocrine pancreas. The induction of expression in the principal islet was Tam dose-dependent. It was possible to achieve a high percentage of labeling in the principal islet (76%) with a high Tam concentration, but we achieved a maximal of only 38% in induced secondary islet cells. A possible reason for this difference is that the *neurod* promotor in the principal islet cells is active by 12 hpf (Flasse et al., 2013) so that more Gal4ER protein has accumulated by the time of Tam treatment as compared to the secondary islet cells, where *neurod* expression follows onset of differentiation triggered by Notch inhibitor treatment. A high toxicity for combining Notch inhibitor LY411575 with Tamoxifen in zebrafish has been reported (Ghaye et al., 2015), we minimized this effect by applying the treatments sequentially, but we were still limited in the maximum Tam dose that we could apply. Similar to the need for confirming functional floxed alleles when using the Cre-loxP system (Carney and Mosimann, 2018; Kirchgeorg et al., 2018), extensive screening was necessary to identify highly responsive UAS lines, and UAS-directed gene expression showed variation between embryos within an experiment. This could result from variability in Tam penetration or efficacy, or uneven Gal4ER activity, as previously described for other inducible Gal4/UAS applications (Hanovice et al., 2016).

As induction of expression from UAS responders can be titrated for low frequency labeling, the Gal4ER/UAS system can be applied to generate mosaic transgene expression. Limitations on maximal expression efficiency render this system less suitable when homogeneous overexpression is required. To overcome the constraints of toxicity and to increase gene expression efficiency, a caged tamoxifen, which can be activated in a specific tissue by UV light, could be used (Faal et al., 2015). This may, in future studies, allow use of higher Tam concentrations and yield more robust gene expression within a defined region. Photocaged versions of Tam have been successfully used in zebrafish in combination with the CreER/loxP and Gal4ER/UAS systems (Sinha et al., 2010; Feng et al., 2017).

Filopodia can probe the environment, prime cell adhesion, as well as mediate intercellular communication which directs tissue morphogenesis (Caviglia and Ober, 2018). Several classes of molecular signals localize to protrusions and can influence morphogenesis through cell-cell contacts. E-cadherin is required in the filopodia that direct preimplantation embryo compaction (Fierro-González et al., 2013). A role for E-cadherin has been demonstrated for clustering of mouse beta cells (Dahl et al., 1996), but a filopodial enrichment of E-cadherin in relation to contact formation has not yet been confirmed. Eph/Ephrin signaling mediates bi-directional signaling during development (Caviglia and Ober, 2018), and localization of components to dendritic filopodial tips influences filopodial behavior during sampling and eventual selection of synaptic targets by neurons (Mao et al., 2018). Expression of Ephs and Ephrins in pancreatic beta cells has been shown to be important for regulating glucose secretion (Jain and Lammert, 2009), however, roles for these proteins in islet formation remain to be determined. Future efforts using our inducible system can define contact-dependent and secreted signals that influence filopodia behavior and direct cell-cell contact formation.

Within the islet, cell-cell communication through paracrine signaling is crucial for fully functional glucose-responsiveness of beta cells (Jain and Lammert, 2009). How cell-cell interactions are established between progenitors during islet development is poorly defined, and having correct connections between transplanted cells is a crucial consideration for islet replacement therapies (Memon and Abdelalim, 2020). Cell-cell contacts originating as cytoplasmic protrusions during islet formation may not arise purely through chance encounters, but may instead be localized to fulfill specific functional roles. The increase of stable filopodia within clustered islet cells suggest these may be direct precursors for structures seen in the mature islet, and represent an enduring morphological feature. We report here long cellular extensions on more differentiated cells expressing sst:GFP, gcga:GFP and ins:GFP in addition to neurod:memKate. Consistent with our findings, dynamic filopodia within the mature islet have been detected on beta cells (Geron et al., 2015) and more recently on somatostatin-secreting delta cells (Arrojo e Drigo et al., 2019), which are suggested to facilitate intra-islet paracrine signaling. While paracrine signaling is expected to occur between directly adjacent cells, cytoplasmic extensions forming signaling contacts between distant cells may be a more general phenomenon that has not been fully appreciated in densely packed islet cells in the absence of sparse cell labeling.

In conclusion, the novel transgenic lines and approaches reported here represent valuable new tools for interrogating molecular mechanisms of pancreatic islet assembly. The mosaic single cell labeling and 3D morphological quantitation that we developed can be applied to studying dynamic cell behaviors during other morphogenetic processes during development, and during pathological processes such as tumor invasion. Furthermore, these studies provide a framework for identification of molecular factors that direct coalescence and formation of the three-dimensional islet structure. Through better understanding of mechanisms regulating pancreatic islet development, crucial signals can be identified and implemented into differentiation protocols to improve the efficiency of generating fully functional islet cells *in vitro* for replacement therapy of diabetes.

## Materials and Methods

### Zebrafish Maintenance and Transgenic Fish Lines

Zebrafish (*Danio rerio*) were maintained according to standard protocols (Kimmel et al., 1995). When necessary to reduce pigmentation, embryos were grown in 0.0015% PTU (Sigma, P7629). For studying juvenile fish, larvae were kept in petri dishes until 5 days post fertilization (dpf), then transferred to our fish facility. Larvae studied between 6 and 9 dpf were kept in a 28°C incubator in petri dishes or 6-well plates and fed with Caviar (5–50 μm; Bern Aqua) once per day.

Transgenic lines used in the study are listed in Supplementary Table S8. The transgenic lines were maintained in a *mitfa* or Tübingen background. Plasmid constructs for newly generated transgenic fish lines were prepared from entry vectors which were combined using Multisite-Gateway Cloning and the Tol2kit (Kwan et al., 2007). The *p5E’-neurod*-*promotor* construct was kindly provided by Alex Nechiporuk (Oregon Health & Science University, United States), *p5E’-6xUAS* by Martin Distel (St. Anna Children’s Cancer Research Institute, Vienna, Austria), *pME-memKate* (ras_mKate2) by Caren Norden (Instituto Gulbenkian Ciência, Lisbon, Portugal), and *pME-Gal4ER* by Scott Stewart (University of Oregon, OR, United States). Generation of *pME-LifeActTdT* was previously described (Freudenblum et al., 2018). *pDest-neurod:Gal4ER* was generated in *pDestTol2CG2* which contains the *cmlc2:EGFP* transgenesis marker, *pDest-UAS:LifeActTdT* contains the *cryaa:tagRFP* transgenesis marker (destination plasmid kindly provided by Wenbiao Chen, Vanderbilt, Tenn, United States). Transgenic lines were generated by injection of 25 ng/μL plasmid DNA and 50 ng/μl transposase mRNA into one-cell stage Zebrafish embryos using standard protocols (Kwan et al., 2007).

*neurod*:*memKate* transgenic lines were examined for pancreatic islet and nervous system expression. Lines with consistent expression and 50% transmission were used for further experiments in embryos and larvae. Results from juvenile fish were obtained using transgenics showing > 50% transmission, and thus with brighter expression presumably from multiple insertions, to better show the details of expression in older animals. The fluorescence from single insertion lines was comparable in location, but dimmer, and difficult to visualize in older samples.

### Heat-Shock (HS) and Compound Treatments

Heat-shock was performed in petri dishes in an air incubator (samples < 4 dpf) or in cell culture flasks placed in a shaking water bath (120 rpm) (samples 4 dpf or older) at 38–39°C for up to two hours. Induction of secondary islet cell differentiation was performed by overnight treatment with Ly411575 (SML0506-5MG, Sigma Aldrich, dissolved in DMSO) at 4 dpf, according to published protocols (Freudenblum et al., 2018).

### Gal4ER Activation by Tamoxifen

Gal4ER was induced by 4-hydroxy-tamoxifen (Tam, H7904-5MG, Sigma Aldrich, dissolved in DMSO) as previously described (Akerberg et al., 2014). To test transgene functionality, *nd:Gal4ER* transgenics were crossed with the *UAS:GFP*, *UAS:mCherry* or *UAS:LifeActTdT* responder lines, with Tam dosing as indicated. Tamoxifen’s relative insolubility and instability in storage leads to variable responses in experiments, even under consistent experimental conditions (Felker and Mosimann, 2016). As we were trying to capture single or sparsely labeled cells in different stages of islet assembly, which itself is a stochastic and variable process, it was difficult to establish an optimal ‘best’ Tam treatment protocol. Therefore, experiments to examine cell morphology and dynamics were performed with a series of Tam treatment conditions (Supplementary Table S2), and samples with bright pancreatic cells, as observed under a dissecting microscope, were selected for subsequent high resolution confocal imaging. In larval samples (5–6 dpf) we achieved robust induction with 1–3 h of 2.5 μM to 25 μM, with lower doses (5 μM) more useful for single cell analyses. For overnight treatments, 2.5 μM was sufficient to activate expression, and larvae tolerated a maximal dose of 5 μM.

### Microscopy

Samples were imaged either live or after a brief fixation as follows: samples were fixed in 4% paraformaldehyde (PFA) in phosphate-buffered saline (PBS) at room temperature for 1 to 2 h, washed three times with 1 x PBS-0.1% Tween, then dissected and embedded in 1.5% low-melting point agarose. Live larvae or juvenile fish were anesthetized with tricaine, settled on a glass-bottom dish and embedded in 1.5% low-melting point agarose overlaid with egg water or E3 medium containing 0.003% tricaine. Confocal fluorescence images were acquired with a Zeiss Axio Observer.Z1 equipped with a Yokogawa CSU-X1 spinning disk using 10x, 25x, 40x or 63x water-immersion lenses or a Zeiss exciter confocal microscope using a 20x water immersion objective lens. For brightfield and widefield fluorescent images, a Leica MZ16FA was used.

### Image Processing and Analysis

To assemble complete images from smaller elements, partially overlapping regions were stitched together using Photoshop (Adobe). Confocal stacks were processed and quantitated in ImageJ/Fiji (Schneider et al., 2012) using plugins as described below. Brightness and contrast adjustment and background subtraction were uniformly applied, and a median filter was applied to reduce speckle noise. Sample shift within a z-stack or time-lapse series was corrected using ‘MultiStackReg’ (Thevenaz et al., 1998) or ‘Correct 3D Drift’ (Parslow et al., 2014). If necessary, single slices were removed from z-stacks if they contained blurring artifacts due to gut contractions. Cell counts based on transgene expression were achieved using the ‘Point Picker’ plugin with confocal z-stacks.

### Single Cell Morphology Analysis

For cell morphology analyses, samples with strong pancreatic expression visible under the dissecting microscope were selected for confocal analysis. An initial image to record cell context included both red and green channels. Subsequent time lapse image series recorded only the red channel, to minimize bleaching and toxicity to the samples. For 3D segmentation of single cells, the initial processing included median filter, background subtraction and contrast enhancement applied uniformly to cropped images. Segmentation of foreground from background was performed using the ‘3D Hysteresis Threshold’ plugin (Ollion et al., 2013). The resulting mask was further processed to fill holes, and smoothed using the ‘Dilate (3D)’ and ‘Smooth (3D)’ functions. Signal not contiguous with the cell of interest was removed using the ‘Purify’ function of ‘BoneJ’ (Doube et al., 2010). Signal from adjacent cells not possible to remove by cropping was trimmed manually in individual slices when necessary. 3D cell parameters were calculated using the ‘Measure 3D’ function of the ‘3D ROI Manager’ (Ollion et al., 2013). Sphericity is a measure of compactness, calculated as a normalized ratio of the object’s surface area to its volume, with a value of 1 representing a perfect sphere. Feret’s diameter, or maximum caliper, is an indicator of cell spreading and is the distance between the two surface pixels located farthest apart.

### Time Lapse Image Analysis

In time lapse movies visualizing cell dynamics, maximum intensity projections are presented as they provide increased signal density and improve visibility of fine protrusions. 3D surface renderings of single cells were generated using the ‘3Dscript’ plugin (Schmid et al., 2019). To measure 3D filopodia length in time lapse movies, the ‘Simple Neurite Tracer’ was used on z-stacks at each time point (Longair et al., 2011), with length values exported to Excel for further analysis. A ‘dynamic event’ is counted when a filopodia (minimum length 1.0 μm) appears, disappears, or changes in length by > 50%. A ‘stable filopodia’ was detected at every time point over the 20 min imaging period and did not change > 50% in length over that time. To determine cell movement trajectories, cells were segmented in 3D using the ‘Segmentation Editor’ and coordinates of cell centers were extracted using the ‘3D ROI Manager’ (Ollion et al., 2013). Cell center coordinates were imported into MATHEMATICA software (Wolfram) to produce a 3D trajectory graph (using a custom written script), in which cells 1–5, 7, and 8 were normalized to cell 6. Cell clustering was quantitated using Matlab as follows: cell-center coordinates determined as described above were exported to Matlab and the volume of a polygon, which contained the segmented cells, was visualized and its volume calculated using the ‘3D convex hull’ function.

### Graphing and Statistics

Graphs and statistical analyses were produced by Prism5 (GraphPad) or OriginPro 2020 (OriginLab). To compare two groups a *t*-test was used. The data is presented as column graphs, dot plots or box plot overlaid with dot plot; in box plots the center line in the box indicates the median, the top and bottom of the box represent interquartile ranges (25 – 75%) and the total ranges are shown in whiskers (0 – 100%); the included dots represent individual data points.

## Data Availability Statement

The datasets generated and analysed during this study are available from the corresponding author upon reasonable request.

## Ethics Statement

All procedures involving animals were approved by the Austrian Bundesministerium fur Wissenschaft und Forschung (GZ BMWFW-66.008/0009-WF/II/3b/2014 and GZ BMWFW-66.008/0018- WF/V/3b/2017) and were carried out in accordance with approved guidelines.

## Author Contributions

JF and RK conceived, designed and performed the experiments, analysed the data, and wrote the manuscript. JF, DM, and RK reviewed and edited the manuscript, and secured funding. DM and RK provided project administration, supervision and resources. All authors contributed to the article and approved the submitted version.

## Conflict of Interest

The authors declare that the research was conducted in the absence of any commercial or financial relationships that could be construed as a potential conflict of interest.
