# Supplementary material for: Inducible Mosaic Cell Labeling Provides Insights Into Pancreatic Islet Morphogenesis
Source: Front Cell Dev Biol. 2020 Sep 25;8:586651. doi: 10.3389/fcell.2020.586651 (PMC7546031; doi:10.3389/fcell.2020.586651)
Supplement: Supplementary file 1 [file Data_Sheet_1.pdf]

SUPPLEMENTARY DATA

This section contains 8 tables, 11 figures and 5 movies.

SUPPLEMENTARY TABLES

Table S1. Related to **Supplementary Figure S4**.

Tam [µM]	Number analysed	Expression at 3h post treatment [%]	Expression at 4h post treatment [%]	Expression at 24h post treatment [%]
**1**	47	18.3	58.7	98.7
**5**	48	31.7	74.0	100.0
**10**	48	48.7	86.0	100.0

Percentage of samples with GFP expression in the principal islet in *nd:Gal4ER;UAS:GFP* transgenics treated at 4 dpf for 1 h with 1 µM, 5 µM or 10 µM Tam.

Table S2. Experimental Design.

Type of Experiment	Tam Treatments	Timing
Single Cell Morphology and Filopodia dynamics<br>Figures 7, 8, S7, S8	5 µM 1 h<br>5 µM 3 h<br>5 µM 3 h + 5 µM o/n	Treatment at 5 dpf or 6 dpf<br>Imaging 24-48 h after treatment
Short interval time lapse<br>Figures 9A, S9A-B	10 µM 1 h & 2.5 µM o/n<br>2.5 µM o/n	Imaging 8-24 h after treatment
Longer interval time lapse<br>Figures 9B, S9C, S10, S11	10 µM x 3 h<br>25 µM x 1 h	Imaging 24 h after treatment

Treatments applied to Tg(*pax6b:GFP;nd:Gal4ER;UAS:LifeActTdT*) triple transgenics following Notch inhibitor treatment at 4 dpf. (h, hours; o/n, overnight)

Table S3. Statistical analysis related to **Figure 7E-H**.

Parameter	Significance	p value	Mean		Standard Deviation	
			iso	clus	iso	clus
**Volume**	*	0.0237	620	464	184	158
**Surface area**	ns	0.0885	795	680	164	181
**Feret's diameter**	ns	0.2425	22	20.5	3.86	2.9
**Sphericity**	ns	0.4064	0.441	0.427	0.0369	0.0452

Analysis by unpaired t-test. (iso, isolated; clus, clustered)

Table S4. Statistical analysis related to **Figure 7I, J.**

Plot		Isolated	Clustered
**Volume vs. Sphericity**	Slope (R^2)	2.4×10^{-5}*# (0.014)	-4.3×10^{-5}*# (0.023)
	Intercept	0.42572	0.44752
**Vol:Ell Ratio vs. Sphericity**	Slope (R^2)	3.30** (0.704)	3.19** (0.765)
	Intercept	-0.790	-0.756

Analysis by linear regression followed by F-test.
[*] The slopes are not significantly different from 0.
[**, #] The two datasets are not significantly different ($p > 0.05$).

Table S5. 2-channel images that correspond to time lapse image series and movies.

Time Lapse Series	2-channel Image
**Figure 8A**	Figure S7A
**Figure 8B**	Figure 7A
**Figure 8C**	Figure S7P
**Figure 8D**	Figure S7N
**Figure S8A**	Figure S7D
**Figure S8B**	Figure S7F
**Figure S8C**	Figure 7D
**Figure S8D**	Figure 7C
**Movie S2**	Figure 7A
**Movie S3**	Figure 7B
**Movie S4**	Figure 7C
**Movie S5**	Figure 7D

Table S6. Statistical analysis related to **Figure 8E,F**.

Parameter	Significance	p value	Mean		Standard Deviation	
			iso	clus	iso	clus
**Dynamic events**	ns	0.9334	10.3	10.0	4.89	7.39
**Stable filopodia**	*	0.0155	0.268	3.57	0.488	3.05

Analysis by unpaired t-test.

Table S7. Summary of protrusion behavior related to **Figure 9A** and **Supplementary Figures S9 & S10**.

Cell-cell dynamics in time lapse movies		No. of occurrences
**Short lived/ exploratory/ briefly connected protrusions**		
	cells connected - not connected	2
	cells not connected - connected - not connected	3
	cells connected - not connected - connected	1
**Stable protrusions associated with cell movements**		
	cells connected - cells move towards each other	3
	cells connected - one cell moves towards the other cell	2

Table S8. Transgenic lines used in this study.

Transgenic line	Abbreviation	Cell type/Compartment	Reference
Tg(neurod:memKate)	nd:memKate	Endocrine, membrane	This work
Tg(neurod:Gal4ER)	nd:Gal4ER	*neurod*-promoter driven Gal4ER	This work
Tg(6xUAS:LifeActTdT)	UAS:LifeActTdT	F-actin labeling	This work
Tg(ela:E2crimson)		Exocrine tissue	(Schmitner et al., 2017)
Tg(P0-pax6b:DsRed)ulg302	pax6b:DsRed	Endocrine, cytoplasm	(Delporte et al., 2008)
Tg(Tp1bglob:EGFP)um14	Tp1:GFP	Notch-responsive cells, cytoplasm	(Parsons et al., 2009)
Tg(P0-pax6b:GFP)ulg515	pax6b:GFP	Endocrine, cytoplasm	(Delporte et al., 2008)
TgBAC(NeuroD:EGFP)nl1	BAC(nd:EGFP)	Early endocrine, cytoplasm	(Obholzer et al., 2008)
Tg(gcga:GFP)	gcga:GFP	Alpha-cells, cytoplasm	(Zecchin et al., 2007)
Tg(Sst2:GFP)	sst2:EGFP	Delta-cells, cytoplasm	(Li et al., 2009)
Tg(UAS:mCherry)		Gal4-responsive expression	(Gift of W. Driever)
Tg(UAS:GFP)		Gal4-responsive expression	(Tessadori et al., 2012)
Tg(Hsp70:Gal4)		Heat-shock induced Gal4	(Hanovice et al., 2016)

SUPPLEMENTARY REFERENCES

Delporte, F.M., Pasque, V., Devos, N., Manfroid, I., Voz, M.L., Motte, P., Biemar, F., Martial, J.A., Peers, B., 2008. Expression of zebrafish pax6b in pancreas is regulated by two enhancers containing highly conserved cis-elements bound by PDX1, PBX and PREP factors. BMC Dev. Biol. 8, 1–19. https://doi.org/10.1186/1471-213X-8-53

Hanovice, N.J., McMains, E., Gross, J.M., 2016. A GAL4-inducible transgenic tool kit for the in vivo modulation of Rho GTPase activity in zebrafish. Dev. Dyn. 245, 844–53. https://doi.org/10.1002/dvdy.24412

Li, Z., Wen, C., Peng, J., Korzh, V., Gong, Z., 2009. Generation of living color transgenic zebrafish to trace somatostatin-expressing cells and endocrine pancreas organization. Differentiation 77, 128–134. https://doi.org/10.1016/J.DIFF.2008.09.014

Obholzer, N., Wolfson, S., Trapani, J.G., Mo, W., Nechiporuk, A., Busch-Nentwich, E., Seiler, C., Sidi, S., Söllner, C., Duncan, R.N., Boehland, A., Nicolson, T., 2008. Vesicular Glutamate Transporter 3 Is Required for Synaptic Transmission in Zebrafish Hair Cells. J. Neurosci. 28, 2110–2118. https://doi.org/10.1523/JNEUROSCI.5230-07.2008

Parsons, M.J., Pisharath, H., Yusuff, S., Moore, J.C., Siekmann, A.F., Lawson, N., Leach, S.D., 2009. Notch-responsive cells initiate the secondary transition in larval zebrafish pancreas. Mech. Dev. 126, 898–912. https://doi.org/10.1016/J.MOD.2009.07.002

Schmitner, N., Kohno, K., Meyer, D., 2017. ptf1a+, ela3l- cells are developmentally maintained progenitors for exocrine regeneration following extreme loss of acinar cells in zebrafish larvae. DMM Dis. Model. Mech. 10, 307–321. https://doi.org/10.1242/dmm.026633

Tessadori, F., Van Weerd, J.H., Burkhard, S.B., Verkerk, A.O., De Pater, E., Boukens, B.J., Vink, A., Christoffels, V.M., Bakkers, J., 2012. Identification and Functional Characterization of Cardiac Pacemaker Cells in Zebrafish. https://doi.org/10.1371/journal.pone.0047644

Zecchin, E., Filippi, A., Biemar, F., Tiso, N., Pauls, S., Ellertsdottir, E., Gnügge, L., Bortolussi, M., Driever, W., Argenton, F., 2007. Distinct delta and jagged genes control sequential segregation of pancreatic cell types from precursor pools in zebrafish. Dev. Biol. 301, 192–204. https://doi.org/10.1016/J.YDBIO.2006.09.041

SUPPLEMENTARY FIGURES

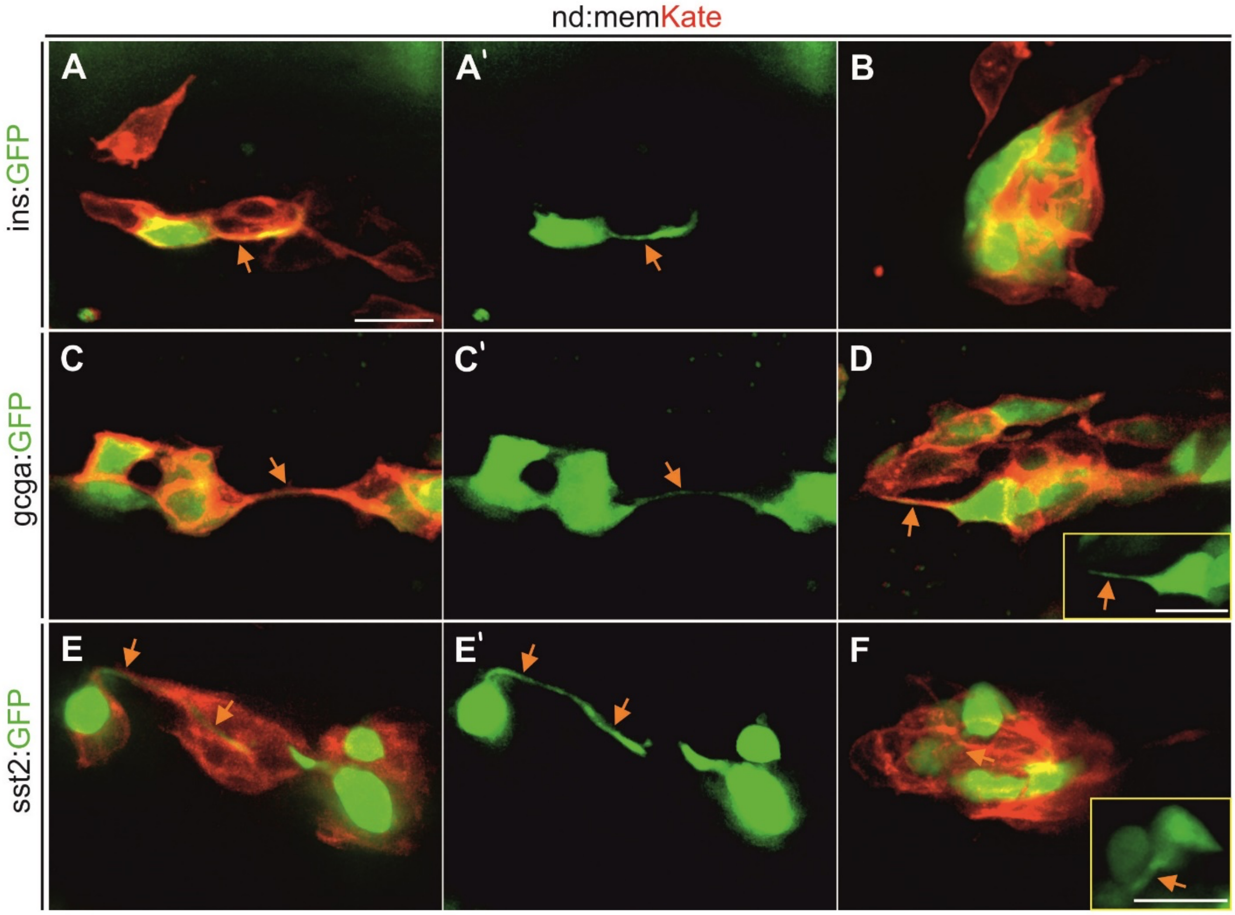

Supplementary Figure S1. Long protrusions displayed by cells coexpressing *neurod;memKate* and endocrine hormones. Endocrine hormones insulin, glucagon and somatostatin are expressed in a subset of neurod:memKate labelled endocrine cells as indicated by transgenes *ins:GFP* **(A-B)**, *gcga:GFP* **(C-D)** and *sst2:GFP* **(E-F)**. Cytoplasmic extensions (orange arrows) extend within (**A, D, F**) and between **(C, E)** cell clusters. (**A', C', E')** Corresponding single-channel GFP images. **D, F**, insets, show single channel substacks highlighting cell protrusions. Scale bars, 10 µm.

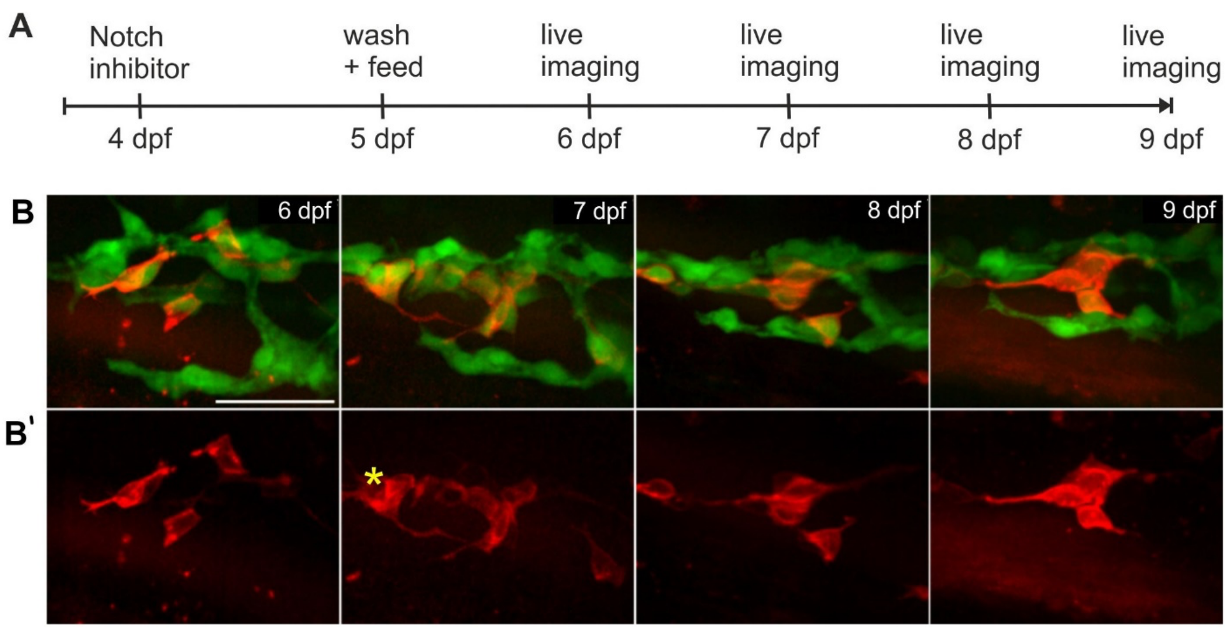

Supplementary Figure S2. Secondary islets assemble at the duct. (A) Timeline of catch-and-release imaging approach. Between 6 and 9 dpf, *Tp1:GFP;nd:memKate* double transgenics were released from agarose into egg water after imaging, and incubated for 24 h at 28°C. Confocal Z-stack projections of posterior pancreas from the stages indicated are shown as a merged view **(B)** and red channel alone **(B')**. The yellow asterisk indicates cells that move out of the field of view. Scale bar, 25 µm.

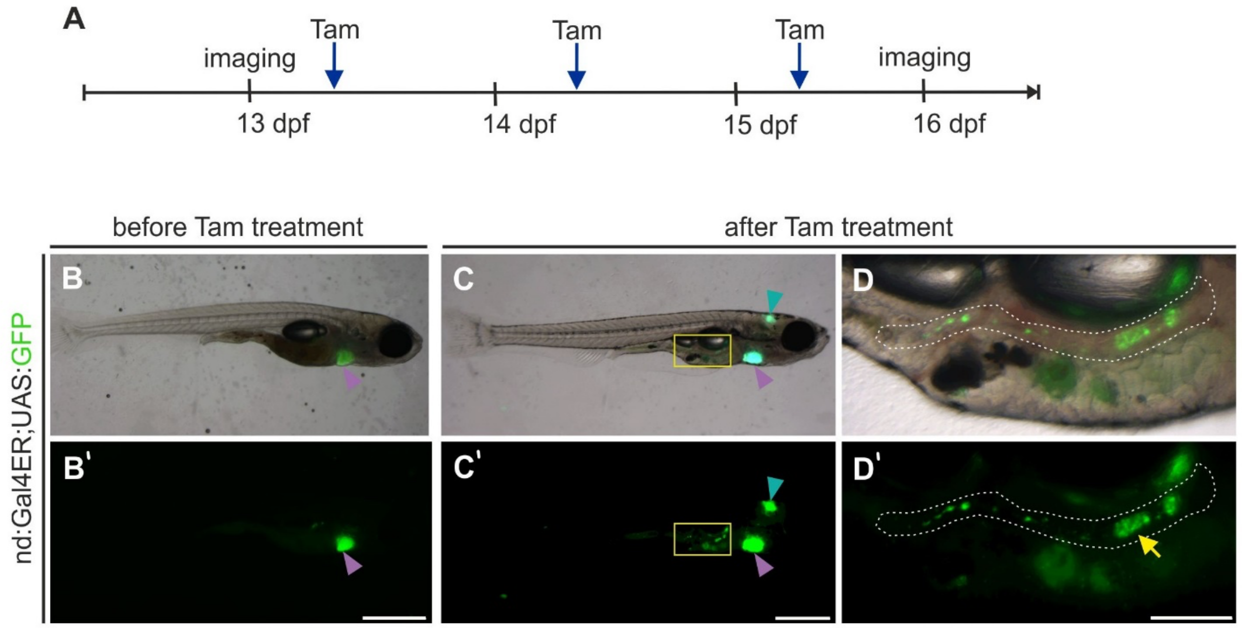

Supplementary Figure S3. Tam-responsive Gal4ER/UAS-system at juvenile stages. (A) Timeline of experiment. Samples were treated for 1 h with 1 µM Tam at 13, 14 and 15 dpf and imaged at 16 dpf. **(B-D)** GFP overlaid with brightfield image. **(B'-D')** GFP image alone. **(B, B')** Prior to Tam treatment, *neurod*-specific GFP expression was not detectable in *nd:Gal4ER;UAS:GFP* double transgenics at juvenile stages. **(C-D)** Following Tam treatment, GFP is expressed in the nervous system **(C, C'**, blue arrowhead indicates forebrain expression) and in the pancreas (**C, C'** yellow box; **D, D'** white outline), visible are the principal (**D'**, yellow arrow) and secondary islets. Region indicated by the yellow box in (**C, C'**) is shown at high magnification in (**D, D'**). Purple arrowhead indicates the heart transgenesis marker. Scale bars: **(B–C)**, 500 µm; (**D)**, 125 µm.

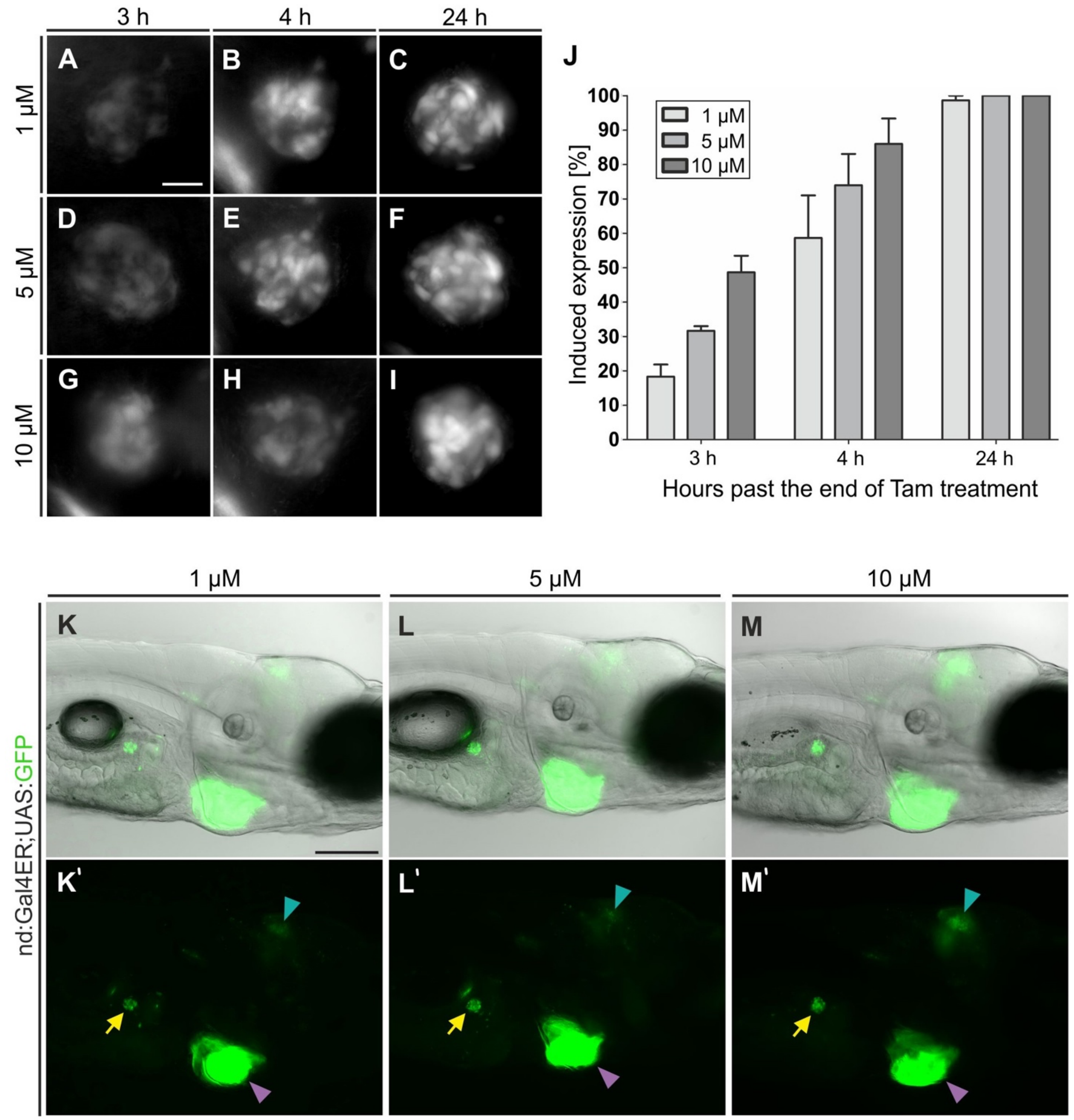

Supplementary Figure S4. Kinetics of *neurod:Gal4ER*-driven transgene expression. (A-I) Principal islets imaged at 3, 4 and 24 hours following Tam treatment for 1 h at the indicated concentrations. Scale bar, 20 µm. **(J)** Quantification of onset of GFP expression relative to Tam concentration, from embryos as shown in **(A-I)**. Shown is percentage (%) of embryos with detectable GFP at the times indicated (see also Supplementary Table S1). **(K-M)** Tam treated *nd:Gal4ER;UAS:GFP* embryos imaged 24 h post treatment, GFP overlaid with brightfield image. **(K'-M')** Corresponding image of GFP alone, indicated is expression in the principal islet (yellow arrow) and brain regions (cyan arrowhead). Purple arrowhead indicates the heart transgenesis marker. Scale bar, 200 µm.

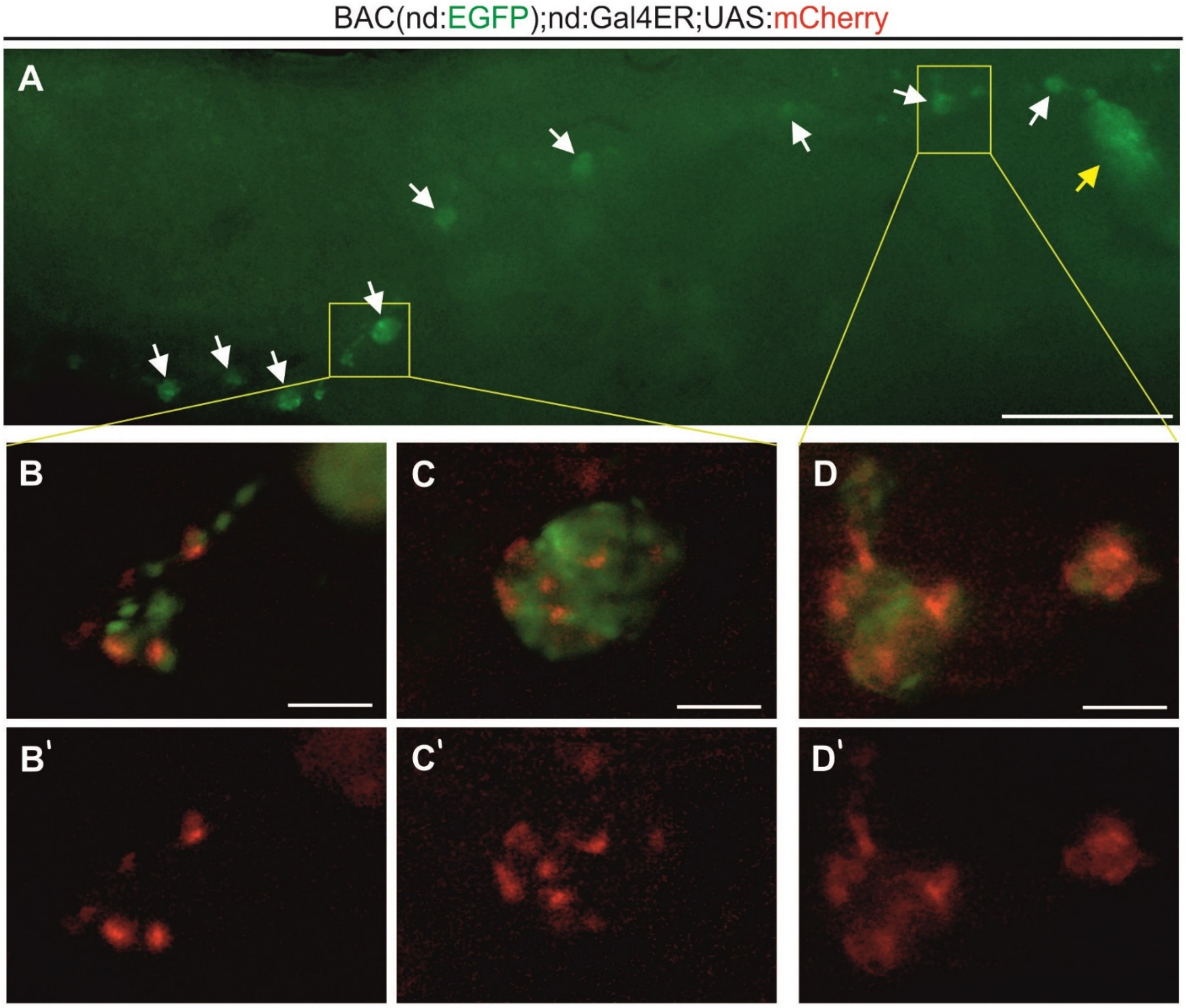

Supplementary Figure S5. Induced transgene expression in secondary islets. (A) GFP expression in a fixed and dissected pancreas from a *BAC(nd:EGFP);nd:Gal4ER;UAS:mCherry* transgenic fish at 8 wpf, following treatment with 1 µM Tam for 1 h per day for three consecutive days. The yellow arrow indicates the principal islet, white arrows indicate secondary islets. **(B-D)** Higher magnification images of secondary islets in regions delineated by yellow boxes, showing overlay of GFP and mCherry **(B, C, D)** and mCherry alone **(B', C', D')**. Scale bars: **(A)**, 200 µm; **(B-D)**, 100 µm.

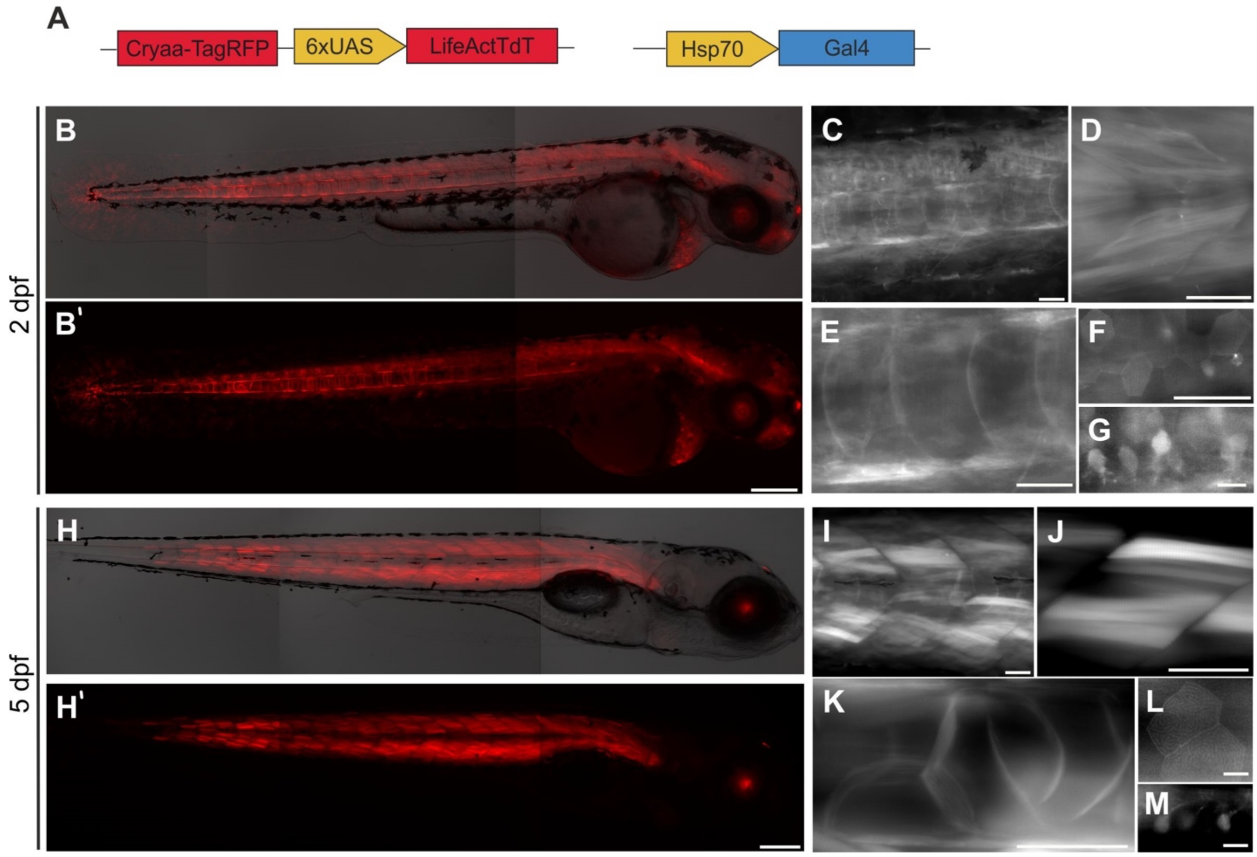

Supplementary Figure S6. Heat-shock induced ubiquitous LifeActTdT expression. (A) Schematic of transgenes for *hsp70:Gal4*-induced expression of *UAS:LifeActTdT*. **(B-M)** *hsp70:Gal4;UAS:LifeActTdT* embryos heat-shocked at 1 dpf, and examined at 2 and 5 dpf (LifeActTdT shown in red). Overlay of LifeActTdT and brightfield image **(B, H)**, and LifeActTdT alone at the indicated stages **(B', H')**. Full images were assembled by stitching together partially overlapping regions. **(C-G, I-M)** Close-ups of LifeActTdT expression (gray) in the trunk **(C, I)**, muscles **(D, J)**, notochord **(E, K)**, skin **(F, L)** and nerve cells **(G, M)**. Scale bars: **(B, H)**, 200 µm; **(C-G, I-M)**, 10 µm.

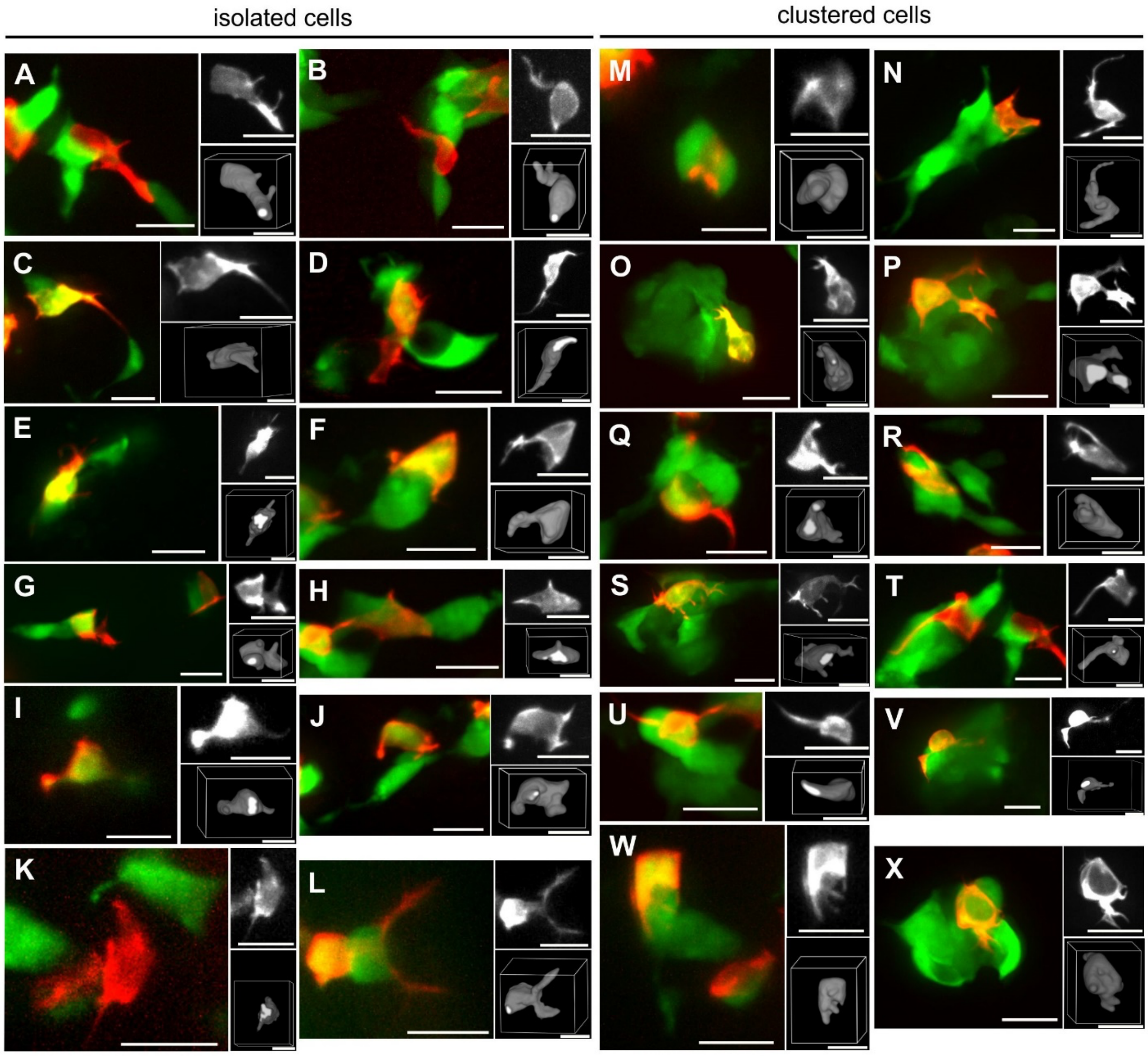

Supplementary Figure S7. Isolated and clustered cells used for 3D-quantification. In *pax6b:GFP; nd:Gal4ER;UAS:LifeActTdT* triple transgenics, all endocrine cells are labelled in green and red/gray shows the Tam-induced LifeActTdT expression. For each isolated **(A-L)** and clustered **(M-X)** cell is shown a z-projection of the 2-channel image (left), a z-projection of the single cell (upper right), and a 3D view of the segmentation mask (lower right). Scale bars, 10 µm.

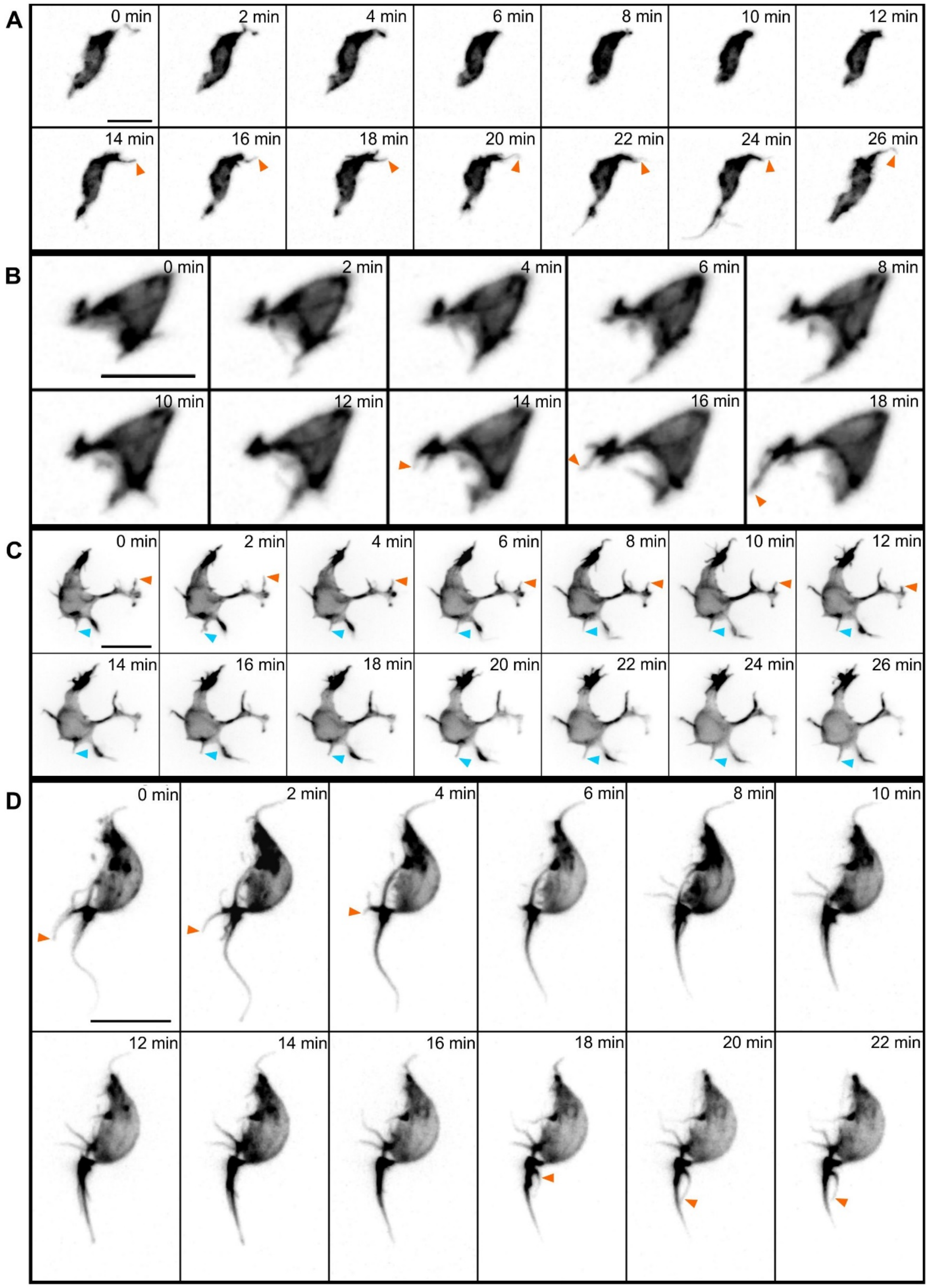

Supplementary Figure S8. Dynamic cell motility of isolated and clustered endocrine cells (related to Figure 8). Z-projections from time lapse image series of pancreatic endocrine cells in *pax6b:GFP; nd:Gal4ER;UAS:LifeActTdT* transgenics (for details see Supplementary Tables S2 and S5). The isolated **(A, B)** and more clustered **(C, D)** samples were imaged at 7 dpf, with images acquired every 2 minutes. The cells show stable (blue arrowheads), as well as dynamic protrusions (orange arrowheads). Scale bars, 10 µm.

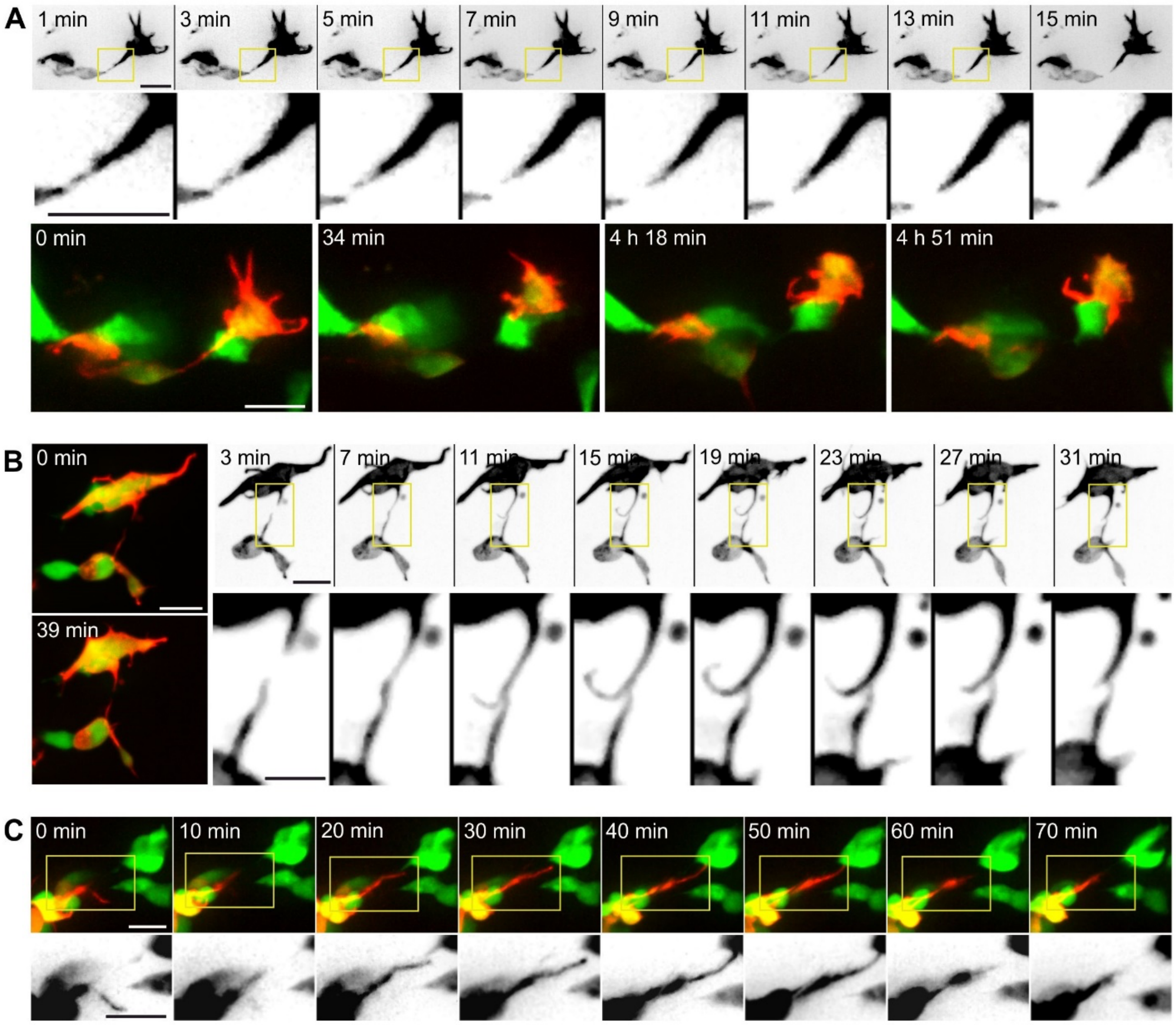

Supplementary Figure S9. Heterogeneous behavior of filopodia extending between secondary islet cells. Image series (as in Figure 9A) showing pancreatic endocrine cells in *pax6b:GFP;nd:Gal4ER; UAS:LifeActTdT* transgenics treated as in Figure 6E and imaged at 6 or 7 dpf, (for details see Supplementary Table S2). Shown are maximum intensity projections at the indicated times (min, minutes), with close-up views of indicated regions (yellow box) show at a higher magnification below, to highlight cell-cell interactions through protrusions (*LifeActTdT*, gray). **(A)** A connection between cells appears to stretch and break apart. **(B)** Filopodia interacting between cells change morphology over a time scale of minutes. **(C)** A connection is maintained over > 10 minutes, then detaches. (See also Supplementary Table S7.) Scale bars, 10 µm.

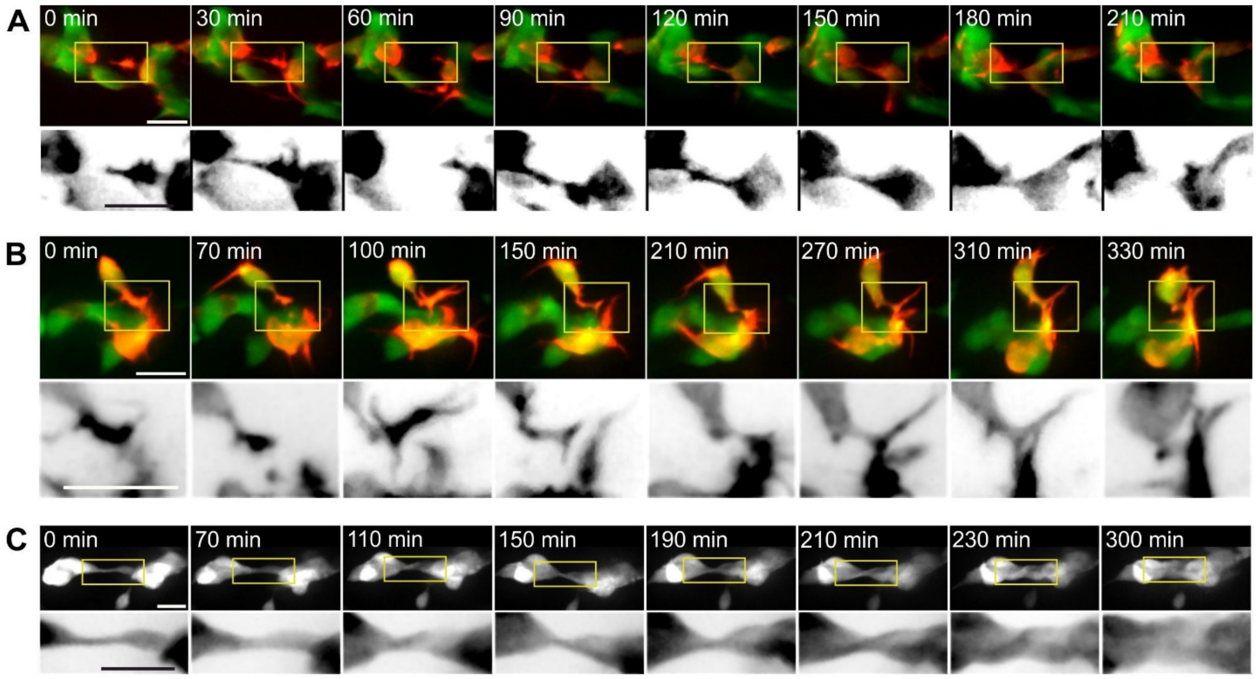

Supplementary Figure S10. Secondary islet cells establish cell-cell contacts via protrusions. (A-C) Image series (as in Supplementary Figure S9, for details see Supplementary Table S2) showing maximum intensity projections of confocal image stacks captured at the indicated times (min, minutes). Close-up views of highlighted regions (yellow box) are shown at a higher magnification in the row below. Cells establish stable connections and move together. (See Supplementary Table S7). Scale bars, 10 µm.

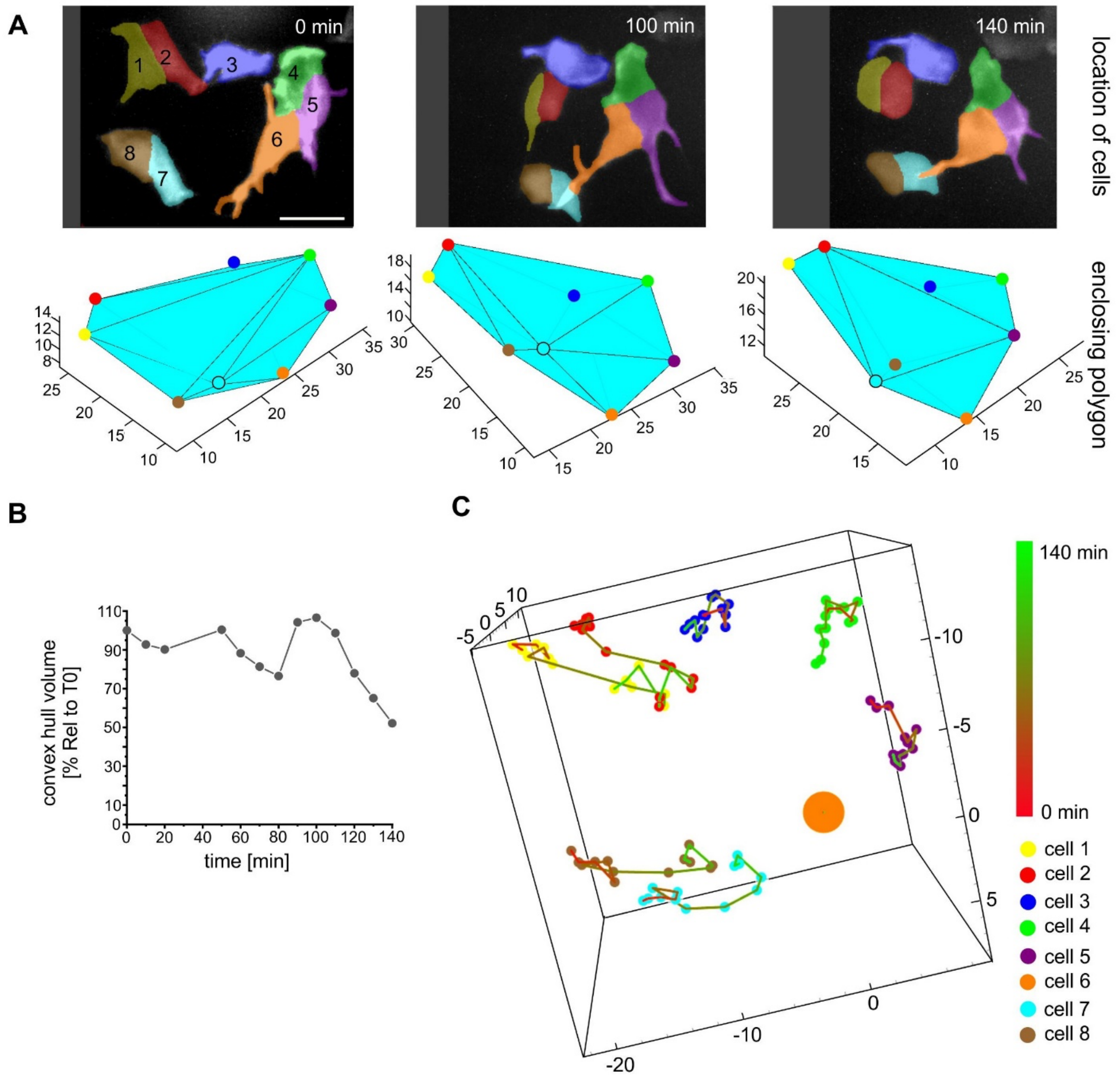

Supplementary Figure S11. Filopodia-based connections bring cells together (related to Figure 9). (A, top) Three time points from the time series shown in **Figure 9B, C,** of the segmented secondary islet cells labelled by pseudo-coloring to facilitate identification**.** An enclosing polygon was determined for each time point **(A, bottom)**. The polygon volume reflects the spread of the cells in 3D and thus provides an index of cell clustering. After 140 minutes, the convex hull volume has decreased to 52% of the value at time 0 **(B)**. **(C)** shows the 3D trajectory graph, in which cells 1-5, 7 and 8 move in relation to the position of cell 6 and the color gradient (red to green) indicates progression of time.

SUPPLEMENTARY MOVIES

Movie S1. Rotation of 3D confocal image stack, generated using Imaris software, showing *Tp1:GFP;neurod:memKate* double transgenic imaged at 2 wpf (Related to **Figure 4**).

Movie S2. Time lapse image series showing dynamic motility of an isolated secondary islet cell (Related to **Figure 7A** & **Figure 8B**).

Movie S3. Time lapse image series showing dynamic motility of an isolated secondary islet cell (Related to **Figure 7B**).

Movie S4. Time lapse image series showing dynamic motility of a secondary islet cell within a cell cluster (Related to **Figure 7C** & **Supplementary Figure S8D**).

Movie S5. Time lapse image series showing dynamic motility of a secondary islet cell within a cell cluster (Related to **Figure 7D** & **Supplementary Figure S8C**).
